# Supplementary material for: Practical N-to-C peptide synthesis with minimal protecting groups
Source: Commun Chem. 2023 Oct 26;6:231. doi: 10.1038/s42004-023-01030-0 (PMC10603086; doi:10.1038/s42004-023-01030-0)
Supplement: Supplementary file 2 — Supplementary Information [file 42004_2023_1030_MOESM2_ESM.pdf]

Supplementary Information

Practical N-to-C peptide synthesis with minimal protecting groups

Toshifumi Tatsumi, Koki Sasamoto, Takuya Matsumoto, Ryo Hirano, Kazuki Oikawa, Masato Nakano, Masaru Yoshida, Kounosuke Oisaki*, Motomu Kanai*

*Correspondence to k.oisaki@aist.go.jp (KO); motomukanai@g.ecc.u-tokyo.ac.jp (MK)

Contents

Supplementary Methods
1. General Methods ········ S2
2. Calculation of HPLC Yield and Epimerization Level ········ S4
3. Synthesis and Screening of Solvents and Additives ········ S5
4. Procedure for N-to-C Peptide Coupling ········ S8
5. Synthesis of Delta-Sleep Inducing Peptide (DSIP) ········ S10
6. Reaction Profiling ········ S15
7. Oxygen Effect ········ S16
8. Sensitivity of PTC toward Hydrolysis ········ S17
9. Preliminary Studies toward Application to Solid Phase Peptide Synthesis ········ S18
10. Analytical Data ········ S19

Supplementary References ········ S54

Supplementary Methods

1. General Method

1-1. General

^1H NMR spectra were recorded on JEOL ECX500 (500 MHz for ^1H NMR and 125 MHz for ^{13}C NMR), JEOL ECS400 (400 MHz for ^1H NMR and 100 MHz for ^{13}C NMR), or Bruker AVANCE III HD 600 (600 MHz for ^1H NMR and 150 MHz for ^{13}C NMR) spectrometer. Chemical shifts were reported in ppm on the δ scale relative to the residual solvent used as an internal reference both for ^1H and ^{13}C NMR.

Low resolution electrospray ionization mass spectra (LRMS, ESI) were measured on Shimadzu LCMS2020 system. High resolution electrospray ionization mass spectra (HRMS, ESI-Q-TOF) were measured on Bruker micrOTOF II or Waters Xevo G3 QTof spectrometer.

Column chromatographies were performed with silica gel 60 (spherical, 40-50 μm, Kanto Chemicals), or packed silica gel columns by Biotage Isolera or Yamazen EPCLC-W-Prep 2XY A type.

Microwave reaction was performed by Biotage Initiator+ (Conditions: Time = 3-6 h, Temperature = 40 ^{o}C, Pressure = off, Pre-stirring = 0 sec, Vial Type = 2.0-5.0 mL or 10-20 mL, Absorption Level = Normal, Fixed Hold Time = On). The temperature was controlled by intermittently irradiating low-level microwave, and the reaction was conducted under primarily thermal conditions.

All non-commercially available compounds (additives) were prepared and characterized as described in Section 3. Other reagents were purchased from Sigma Aldrich, Tokyo Chemical Industry Co., Ltd. (TCI), Kanto Chemical Co., Inc., FUJIFILM Wako Pure Chemical Co., Peptides Institute, Inc., Watanabe Chemical Industries, Ltd., or Nacalai Tesque, Inc. and used without further purification. Water was purified using a Merck Millipore Milli-Q water purification system.

1-2. Analytical HPLC

Analytical HPLC data were acquired using a JASCO HPLC system equipped with UV-2075 spectrometer, PU-2080 pumps, AS-2055 Plus autosampler, DG-2080-54 degasser, and MX-2080-32 dynamic mixer.

Analytical HPLC was performed using a C18 reversed-phase column (150 mm × 4.6 mm I.D., YMC-Triart C18) with A: 0.1% TFA aq., B: MeCN eluent system. The eluent was monitored by the absorbance at 230 nm.

Analytical HPLC was performed by one of the following methods.

method A: flow rate 1 mL/min, B/A = 0% over 0-2 min, 0-100% over 2-42 min (linear gradient), 100% over 42-62 min, 230 nm

method B: flow rate 1 mL/min, B/A = 0% over 0-2 min, 0-100% over 2-82 min (linear gradient), 100% over 82-102 min, 230 nm

method C: flow rate 1 mL/min, B/A = 0% over 0-2 min, 0-100% over 2-42 min (linear gradient), 230

nm

1-3. Analytical chiral HPLC for determination of epimerization levels of PTCs

The epimerization levels of PTCs were determined by chiral HPLC analysis conducted by a JASCO HPLC system (pump: PU-2080; detector: UV-2075, measured at 254 nm; chiral column; mobile phase: hexane/2-propanol).

1-4. Preparative HPLC

Preparative HPLC was conducted by using a JASCO HPLC system equipped with UV-2075 plus spectrometer, PU-4086 pump, DG-4580 degasser, MX-2080-32 dynamic mixer, CO-4065 column oven, and CHF122SC fraction collector. Collected desired fractions were lyophilized using EYELA FDU-2110 machine.

Preparative HPLC was performed using a C18 reversed-phase columns (250 × 20 mm I.D., YMC-Triart Actus C18) with A: 0.1% TFA aq., B: MeCN eluent system. Temperature of column oven was set at 40 °C. The eluent was flowed at 10 mL/min and monitored by the absorbance at 230 nm. Gradient time program was set as follows.

method D: B/A = 20% over 0-5 min, 20-80% over 5-95 min (linear gradient), then wash with 100% B.

1-5. LC-MS analysis

LC-MS analysis was performed by Shimadzu LCMS2020 coupled with Prominence-I LC-2030C system, and N_2 supplier model 24F.

LC was performed using a C18 reversed-phase column (150 × 4.6 mm I.D., YMC-Triart C18) at room temperature with A: 0.1% HCO_2H aq., B: MeCN eluent system. The eluent was monitored by the absorbance at 230 or 254 nm.

LC-MS was performed with one of the following methods.

method E: flow rate 0.6 mL/min, linear gradient B/A =2% over 0-1 min, 2-100% over 34 min, 100% for 5 min, 254 nm

method F: flow rate 0.6 mL/min, linear gradient B/A = 2% over 0-1 min, 2-100% over 34 min, 100% for 5 min, 230 nm

method G: flow rate 1.0 mL/min, linear gradient B/A = 2% over 0-1 min, 2-100% over 30 min, 100% for 19 min, 254 nm

method H: flow rate 1.0 mL/min, linear gradient B/A = 2% over 0-1 min, 2-100% over 30 min, 100% for 19 min, 230 nm

method I: flow rate 0.6 mL/min, linear gradient B/A = 2% over 0-1 min, 2-60% over 20 min, 100% for 5 min, 254 nm

2. Calculation of HPLC Yield and Epimerization Level

2-1. Calculation of HPLC yield

HPLC yield was determined from the corresponding calibration curve between the peak area (absorbance at 230 nm) of the product and the molarity using an analytical HPLC. For preparing the calibration curve, an authentic peptide was dissolved in DMSO to make a 20 mM peptide solution and the solution was diluted to 15, 10, 5, 2.5, and 2 mM except otherwise noted. For peptides containing an Arg(Pbf), a His(Trt), or a Tyr residue, or a Fmoc group, calibration curves were prepared using 2, 1, 0.5, 0.25, and 0.125 mM concentrations. For peptides containing a Trp residue, calibration curves were prepared using 0.5, 0.25, 0.125, 0.0625 mM concentrations.

2-2. Calculation of epimerization level for PTC 1

Following our previous protocol,[1] the epimerization level of PTCs **1** (TEL) was calculated after conversion to a *p*-methoxybenzyl thioester (peptide-SPMB) by the treatment with *p*-methoxybenzyl chloride. The reaction mixture containing a thioester was analyzed by normal-phase chiral HPLC. The peak areas of the LL / LD isomers were measured and the epimerization level was calculated based on the following equation.

$$\text{epimerization level for PTC (TEL)}[\%] = \frac{\text{peak area (LD)}}{\text{peak area (LD)} + \text{peak area (LL)}} \times 100$$

2-3. Calculations of epimerization level after coupling reaction

Epimerization levels of coupling reaction were calculated as follows.

After peptide coupling reaction, the reaction mixture was analyzed by reverse phase HPLC. The peak areas corresponding to LLL isomers and LDL isomers were measured, and the apparent epimerization level (AEL) was calculated based on following equation.

$$\text{apparent epimerization level (AEL)}[\%] = \frac{\text{peak area (LDL)}}{\text{peak area (LDL)} + \text{peak area (LLL)}} \times 100$$

This AEL value indicates the epimerization level of substrate PTC for the peptide coupling reaction. The epimerization level of the peptide coupling reaction was calculated based on the following equation.

$$\text{epimerization level }[\%] = \frac{(100-\text{TEL}) - (100-\text{AEL})}{100 - 2 \times \text{TEL}} \times 100$$

3. Synthesis and Screening of Solvents and Additives

3-1. Synthesis of HOPO^{Me}

HOPO^{Me} was synthesized by following the reported procedure.[2]

3-2. Synthesis of HOPO^{Phy}

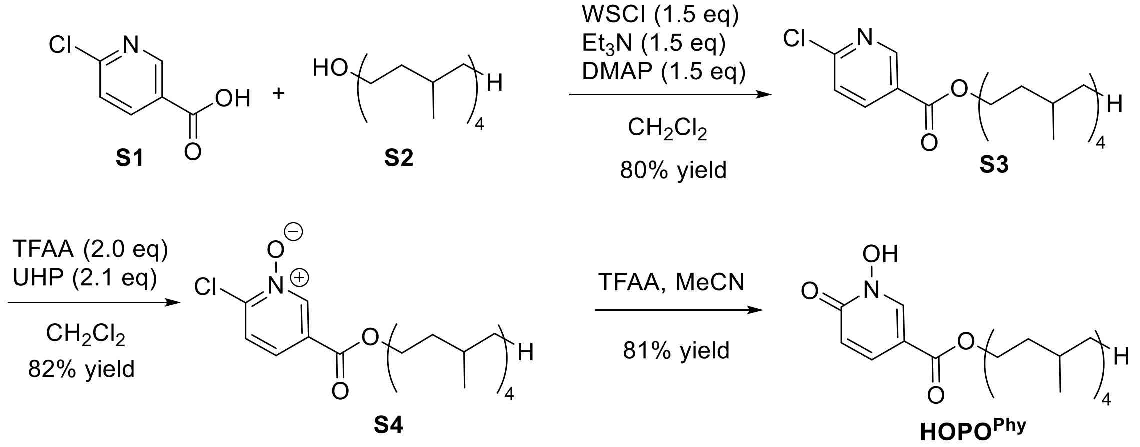

Step 1: To a mixture of **S1** (4 g, 25.2 mmol), WSCI (7.2 g, 37.8 mmol, 1.5 equiv), and DMAP (4.6 g, 37.8 mmol, 1.5 equiv) in CH_2Cl_2 (80 mL), 3,7,11,15-tetramethylhexadecan-1-ol (**S2**, 10.8 mL, 30.2 mmol, 1.2 equiv) and triethylamine (7.2 mL, 37.8 mmol, 1.5 equiv) were added at room temperature. The mixture was stirred for 25 hours at room temperature under an argon atmosphere. After completion of the reaction, solvents were removed under reduced pressure. Ethyl acetate and 1 M HCl aq. were added to the residue, and the mixture was extracted with ethyl acetate. The combined organic layers were washed with satd. $NaHCO_3$ aq (twice), brine, and dried over Na_2SO_4. After filtration, volatiles were removed under reduced pressure to afford crude product. The crude mixture was purified by column chromatography (silica gel, hexane/ethyl acetate = 80:20 → 60:40) to give **S3** (8.85 g, 80% yield).

Step 2: To a solution of **S3** (8.85 g, 20.2 mmol) and urea hydrogen peroxide (UHP, 3.95 g, 42.4 mmol, 2.1 equiv) in CH_2Cl_2 (50 mL), trifluoroacetic acid anhydride (TFAA, 5.7 mL, 40.4 mmol, 2.0 equiv) was added dropwise at 0 °C and the mixture was stirred at room temperature for 16 hours under argon atmosphere. The mixture was cooled to 0 °C. Then, satd. $NaHSO_4$ aq. (5.5 mL) was added to the mixture. The resulting mixture was extracted with ethyl acetate. The combined organic layers were washed with satd. $NaHCO_3$ aq. (twice), brine, and dried over Na_2SO_4. After filtration, volatiles were removed under reduced pressure to afford crude product. The crude mixture was purified by column chromatography (silica gel, hexane/ethyl acetate = 90:10 → 0:100) to give **S4** (7.51 g, 82% yield).

Step 3: To a stirred solution of **S4** (7.51 g, 16.5 mmol) in MeCN (15 mL), TFAA (30 mL) was added

at room temperature and the mixture was stirred for 14 hours under argon atmosphere. The solvent was removed under reduced pressure and solid $NaHCO_3$ and $CHCl_3$ were added to the residue. After filtration, volatiles were removed under reduced pressure to give crude product. The crude mixture was purified by column chromatography (neutral silica gel, hexane/ethyl acetate = 60:40 → 0:100) to give **HOPO^{Phy}** as yellow oil (5.82 g, 81% yield).

Spectral data for **HOPO^{Phy}**:
^{1}H NMR (391.8 MHz, $CDCl_3$): δ 9.38 (brs, 1H), 8.53 (d, *J* = 2.2 Hz, 1H), 7.95 (dd, *J* = 2.2 Hz, *J* = 9.4 Hz, 1H), 6.69 (d, *J* = 9.4 Hz, 1H), 4.31 (m, 2H), 1.75 (m, 1H), 1.54 (m, 3H), 1.0-1.4 (m, 20H), 0.92 (d, *J* = 6.3 Hz, 3H), 0.82 (m, 12H);
^{13}C NMR (98.5 MHz, $CDCl_3$): δ163.5, 159.1, 138.2, 136.5, 136.4, 117.2, 110.9, 64.2, 39.5, 37.6, 37.5, 37.5, 37.4, 37.4, 37.4, 37.3, 35.7, 35.6, 32.9, 32.9, 30.0, 28.1, 24.9, 24.6, 24.4, 22.8, 22.7, 19.9, 19.8, 19.7, 19.7, 19.6;
HRMS (ESI) *m/z*: $[M+Na]^+$ calcd. for $C_{26}H_{45}NNaO_4$, 458.3241; found 458.3224.

<u>3-3. Screening of greener solvents</u>

Because toluene is not an ideal solvent from a green chemistry perspective,[3] we studied other solvent systems. The following results showed DMSO/toluene (1:1) as the best solvent.
Analytical HPLC: method C.

Table S1. Screening of greener solvents

Cbz-Phe-Val-SH **1b** 50 mg → [H-Ala-OH (2.0 equiv), HOPO^{Me} (2.0 equiv); DMSO/Solvent (1:1, 100 mM), 30 °C, 6 h, air] → Cbz-Phe-Val-Ala-OH **2ba**

Entry	Solvent	HPLC yield (%)	epi. level (%)
1	DMSO/toluene	76	< 1
2	DMSO/AcOEt	74	1
3	DMSO/AcO^{i}Pr	72	< 1
4	DMSO/CPME	79	1.2
5	DMSO/IPA	61	1.2
6	DMSO/toluene (10:1)	77	3.6

3-4. Screening of HOPO additives

HOPO$^{\mathbf{Phy}}$ was selected as the best additive on the basis of the following results.

Table S2. Screening of HOPO additives

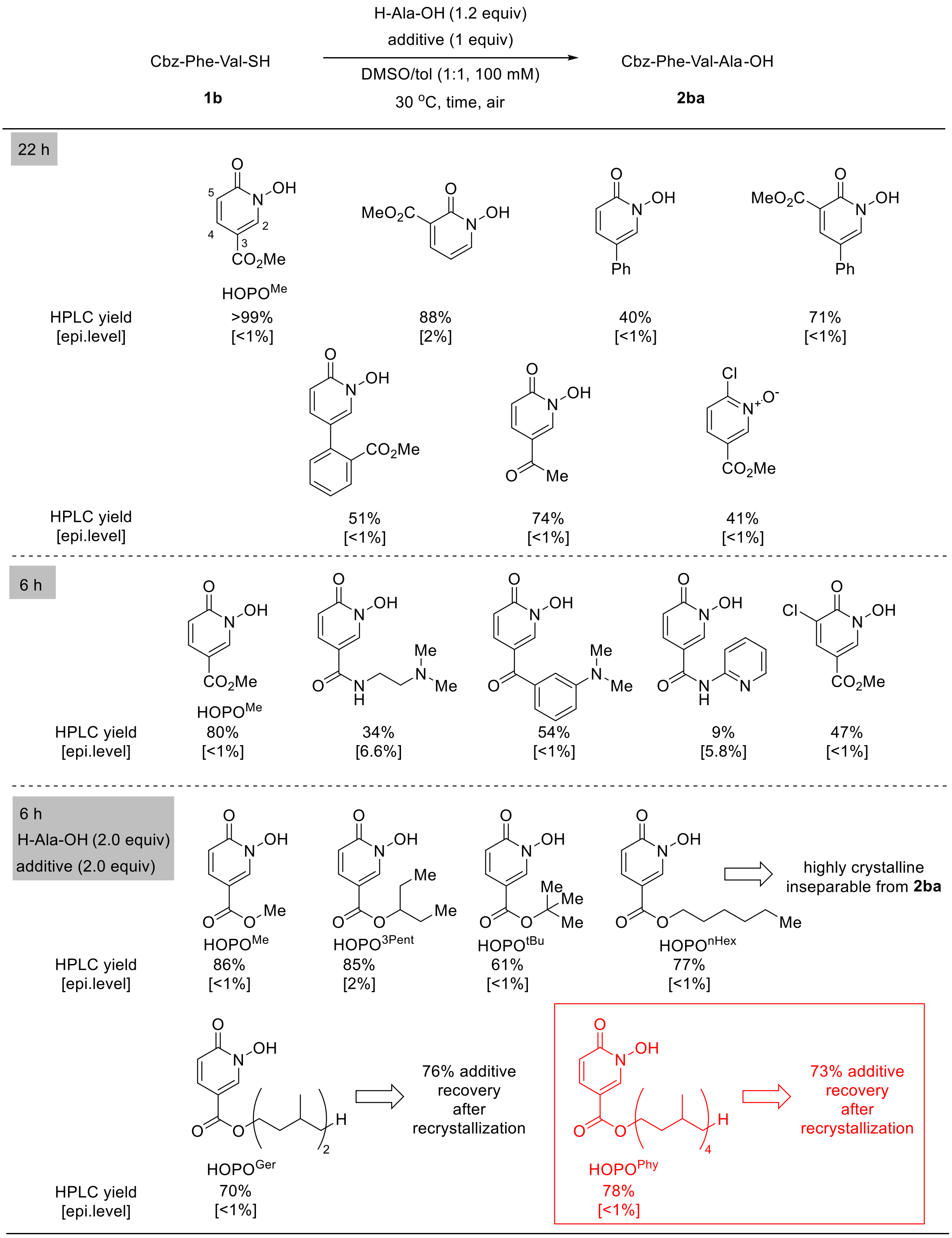

4. Procedure for N-to-C Peptide Coupling

4-1. General procedure for the synthesis of PTC (represented by synthesis of 1b)

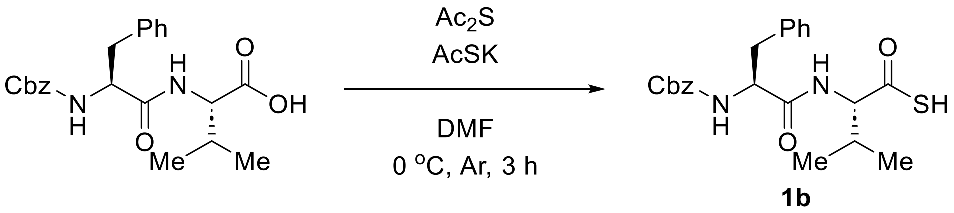

Each PTC was synthesized by following the reported procedure[1] with slight modifications. Cbz-Phe-Val-OH (3.74 g, 9.39 mmol), potassium thioacetate (10.7 g, 93.9 mmol, 10 equiv) and DMF (94 mL) were mixed under argon atmosphere. To this solution, diacetyl sulfide (197 μL, 1.88 mmol, 0.2 equiv) was added at 0 °C under argon atmosphere. After stirring for 5.5 hours at 0 °C, ethyl acetate, water, 1 N HCl aq. were added at 0 °C. The mixture was extracted with ethyl acetate. Combined organic layers were washed with 1 N HCl aq., water, and brine, and dried over Na_2SO_4. After filtration, volatiles were removed under reduced pressure to afford crude product. The crude mixture was purified by silica gel column chromatography (the crude sample was mounted on pre-cooled silica gel by dry ice, hexane/ethyl acetate = 20:80 → 70:30) to give Cbz-Phe-Val-SH (**1b**) as brown solid (2.70 g, 69% yield). Epimerization level was determined as 5.2% by following the protocol described in Section 2-2.

4-2. General procedure for N-to-C peptide coupling (represented by the synthesis of 2bb)

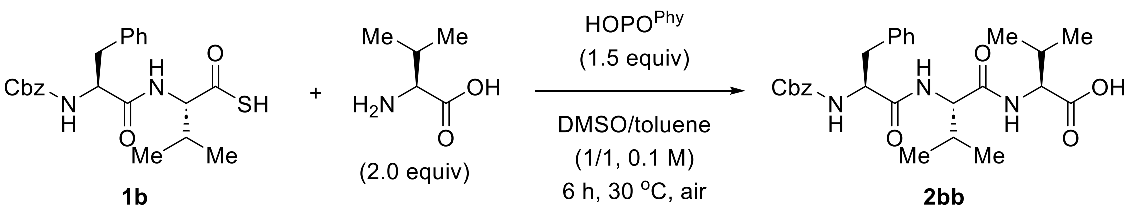

Cbz-Phe-Val-SH (**1b**, 50 mg, 0.12 mmol, 5.2% epi. level), $HOPO^{Phy}$ (78.4 mg, 0.18 mmol, 1.5 equiv), and L-valine (28.1 mg, 0.24 mmol, 2.0 equiv) were added to a test tube. Toluene (600 μL) and DMSO (600 μL) were then added, and the reaction mixture was stirred at 30 °C for 6 hours under air. A HPLC sample was prepared by diluting 12 μL of the reaction mixture with 68 μL of 1% TFA/DMSO. The HPLC yield was determined as 99% and the epimerization level was calculated as <1% (method A). Volatiles were removed under reduced pressure, then ethyl acetate and 1 M HCl aq. were added to the residue and products were extracted with ethyl acetate. The combined organic layeres were dried over Na_2SO_4. After filtration, volatiles were removed under reduced pressure to afford the crude tripeptide. The crude mixture was purified by column chromatography (silica gel, hexane/ethyl acetate = 70:30 → 0:100) to give tripeptide **2bb** as white cotton (50 mg, 84% isolated yield).

4-3. Procedure for peptide fragment coupling (synthesis of 2br)

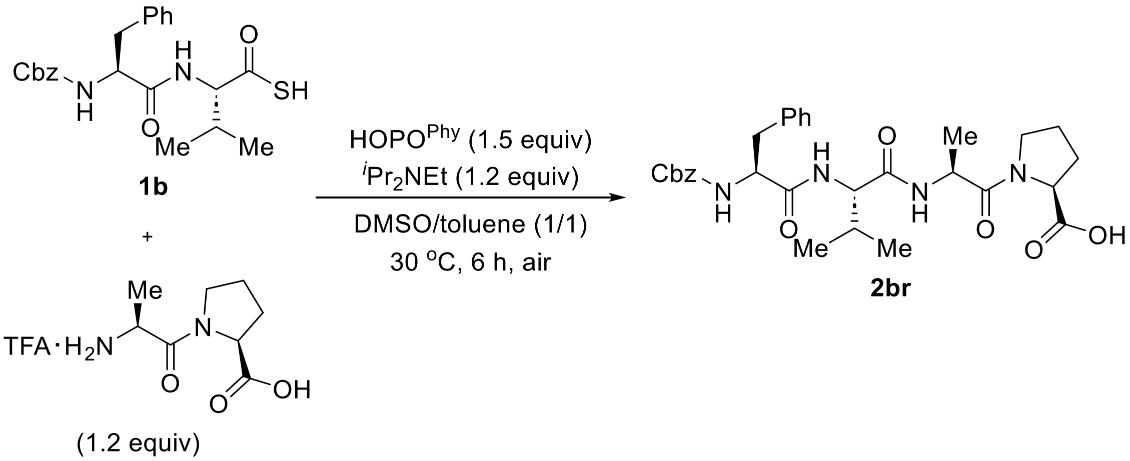

To a solution containing Cbz-Phe-Val-SH (**1b**, 50 mg, 0.12 mmol, 5.2% epi. level), H-Ala-Pro-OH TFA salt (43 mg, 0.144 mmol, 1.2 equiv), and HOPO^{Phy} (78.4 mg, 0.18 mmol, 1.5 equiv) in DMSO (600 μL) and toluene (600 μL), $^{i}Pr_2NEt$ (25 μL, 0.144 mmol, 1.2 equiv) was added. After stirring at 30 °C for 6 hours, a HPLC sample was prepared (12 μL of the reaction mixture was mixed with 68 μL of 1% TFA/DMSO). HPLC yield was determined as 99% (method B).

TFA (68 μL) was added to the reaction mixture to quench the reaction. Ethyl acetate and 1 M HCl aq. were added to the resulting mixture, and organic compounds were extracted with ethyl acetate (three times). The combined organic layers were washed with brine and dried over Na_2SO_4. After filtration, volatiles were removed under reduced pressure. The crude mixture was purified by column chromatography (silica gel, hexane/ethyl acetate = 80:20, then chloroform/methanol = 100:0 → 80:20). The obtained material was further purified by reverse-phase preparative HPLC (method C, t_R = 52.0 min). Fractions containing the desired product were combined and lyophilized to afford analytically pure **2br** (43.3 mg, 64% isolated yield).

5. Synthesis of Delta-Sleep Inducing Peptide (DSIP)

5-1. Procedure for a gram-scale N-to-C peptide coupling (represented by the synthesis of Fragment 1)

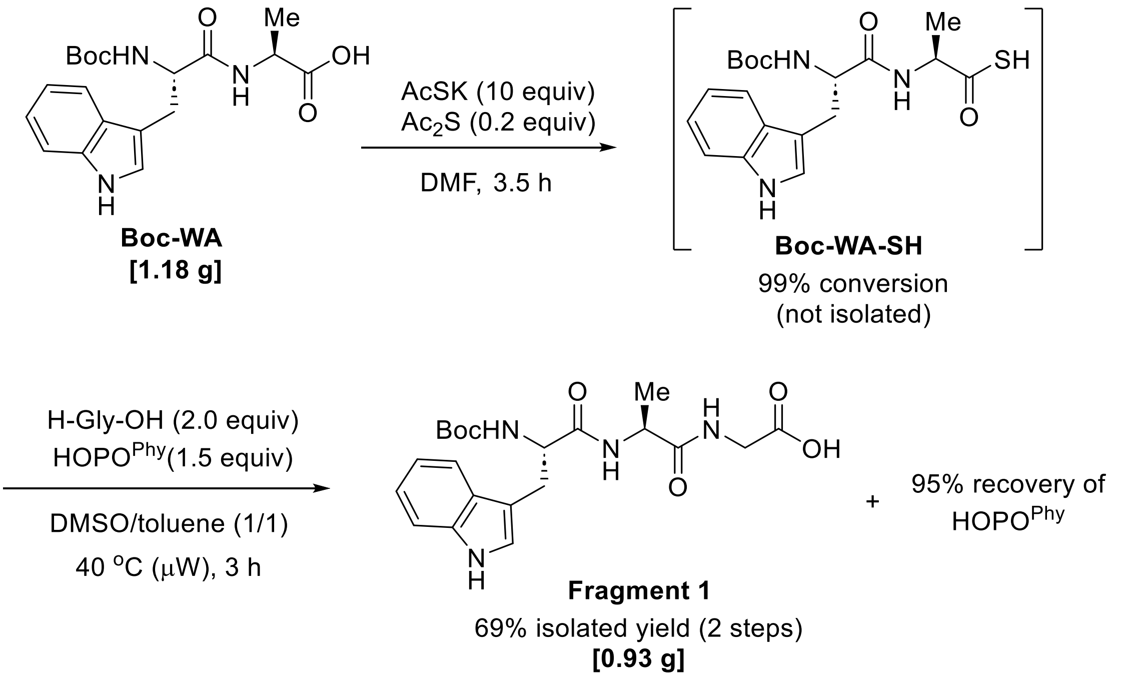

To a mixture of **Boc-WA** (1.18 g, 3.14 mmol), potassium thioacetate (3.59 g, 31.4 mmol, 10 equiv) and DMF (31.4 mL), diacetyl sulfide (65.7 μL, 0.628 mmol, 0.2 equiv) was added dropwise at 0 °C and the mixture was stirred for 3.5 hours at 0 °C under argon atmosphere. Ethyl acetate, water, and 1 M HCl aq. were added to the reaction mixture and products were extracted with ethyl acetate. The combined organic layers were washed with water, 1 M HCl aq., and brine, dried over Na_2SO_4, and filtered. Volatiles were removed under reduced pressure to afford crude PTC (**Boc-WA-SH**). This crude product was used for the next reaction without further purification.

Boc-WA-SH (estimated as 3.14 mmol) dissolved in DMSO (6.5 mL) was added to a mixture of glycine (471 mg, 6.18 mmol, 2.0 equiv), HOPO^{Phy} (2.05 g, 4.71 mmol, 1.5 equiv), and toluene (6.5 mL) in a test tube for microwave irradiation. The mixture was stirred in a microwave apparatus at 40 °C for 3 hours. Ethyl acetate, water, and 1 M HCl aq. were added to the reaction mixture, and the mixture was extracted with ethyl acetate. The combined organic layers were washed with brine, dried over Na_2SO_4, filtered, and volatiles were removed under reduced pressure. Hexane (150 mL) was added to the crude product and the mixture was sonicated. The peptide was filtered out while washing with hexane. Volatiles of the filtrates were removed under reduced pressure and crude HOPO^{phy} was recovered. This was further purified by column chromatography (neutral silica gel, hexane/ethyl acetate = 80:20 → 50:50) to afford HOPO^{phy}, which was reusable for another reaction (1.95 g, 95% recovery).

The crude mixture on the filter containing tripeptide was dissolved into a large amount of methanol

and ethyl acetate, and the solvent was removed under reduced pressure. Ethyl acetate (50 mL) and activated carbon (400 mg) were added to the mixture. This suspension was stirred at 80 °C for 10 min under an argon atmosphere and then cooled to room temperature. The suspension was filtered over Celite and washed with ethyl acetate. Volatiles of the filtrate were removed under reduced pressure to afford the sulfur-removed tripeptide. This crude product was further purified by column chromatography (silica gel, hexane/ethyl acetate = 70:30 → 0:100 then ethyl acetate/methanol = 90:10 → 80:20) to afford tripeptide **Fragment 1** (931 mg, 69%).

5-2. Procedure for Fmoc removal (represented by the synthesis of Fragment 2)

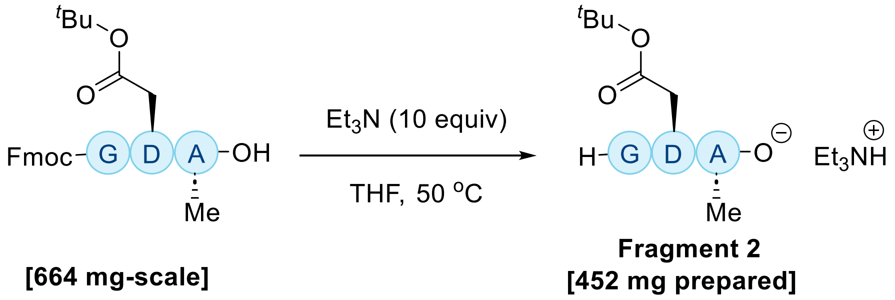

To a solution of Fmoc-GD(ᵗBu)A-OH (664 mg, 1.23 mmol) in THF (24.6 mL), triethylamine (1.71 mL, 12.3 mmol, 10 equiv) was added at room temperature under an argon atmosphere. The mixture was heated at 50 °C and stirred for 22 hours. The reaction mixture was cooled to room temperature and volatiles were removed under reduced pressure. Diethyl ether was added to the residue and water-soluble compounds were extracted with water (three times). The combined aqueous layer was concentrated using evaporator and then lyophilizer. The obtained crude **Fragment 2** (452 mg, pale yellow amorphous solid) was used for the next fragment coupling reaction without further purification.

5-3. Procedure for subgram-scale fragment coupling (synthesis of hexapeptide 3)

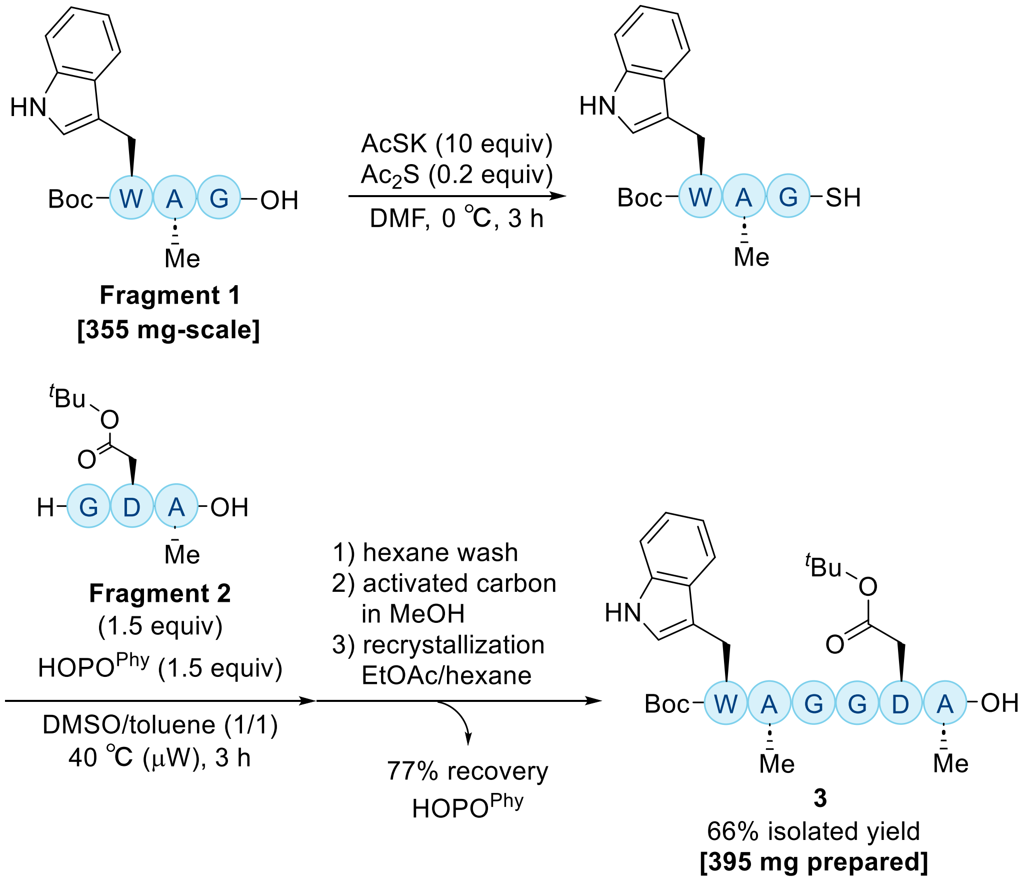

To a mixture of Boc-WAG-OH (**Fragment 1**, 355 mg, 0.820 mmol) and potassium thioacetate (936 mg, 8.20 mmol, 10 equiv) in DMF (8.2 mL), diacetyl sulfide (17.1 μL, 0.164 mmol, 0.2 equiv) was added at 0 °C under an argon atmosphere. After stirring for 3 hours at 0 °C, ethyl acetate, water, and 1 N HCl aq. were added in this order. The organic compounds were extracted with ethyl acetate (three times). The combined organic layers were washed with water, 1 N HCl aq., and brine, and then dried over Na_2SO_4. Volatiles were removed under reduced pressure to afford Boc-WAG-SH. The crude product was used in the next step without further purification.

To a solution of crude H-GD(^{t}Bu)A-OH (**Fragment 2**, 452 mg, 1.23 mmol, 1.5 equiv) and HOPO^{Phy} (536 mg, 1.23 mmol, 1.5 equiv) in DMSO (2.5 mL) and toluene (2.5 mL), Boc-WAG-SH (0.82 mmol) in DMSO (2.5 mL) and toluene (2.5 mL) was added at room temperature under air. After stirring at 40 °C for 3 hours using a microwave apparatus, 1 N HCl aq. and water were added to the reaction mixture. The organic compounds were extracted with ethyl acetate (three times), and the combined organic layers were washed with brine, then dried with Na_2SO_4. To the obtained crude mixture, hexane (150 mL) was added and the mixture was subjected to sonication. The precipitate containing the desired hexapeptide was collected by filteration while washing with hexane. The filtrate was concentrated and subjected to column chromatography (neutral silica gel, hexane/ethyl acetate = 80:20

→ 30:70) to recover HOPO^{Phy} (415 mg, 77% recovery).

The filtered crude product containing the hexapeptide was dissolved in methanol (50 mL) and activated carbon (500 mg) was added. The mixture was heated with stirring at 80 °C for 10 min. The mixture was then cooled to room temperature and filtered through Celite pad while washing with methanol. After removal of the solvent from the filtrate under reduced pressure, the obtained crude product was recrystallized with ethyl acetate/hexane. The precipitated crystals were collected by filtration while washing with ethyl acetate/hexane (1/1). The collected compound was dissolved into ethyl acetate/methanol and evaporated. To the residue, water and acetonitrile were added and the solution was subjected to lyophilization to give hexapeptide **3** as white solid (395 mg, 66% yield).

5-4. Procedure for subgram-scale fragment coupling (synthesis of nonapeptide 4)

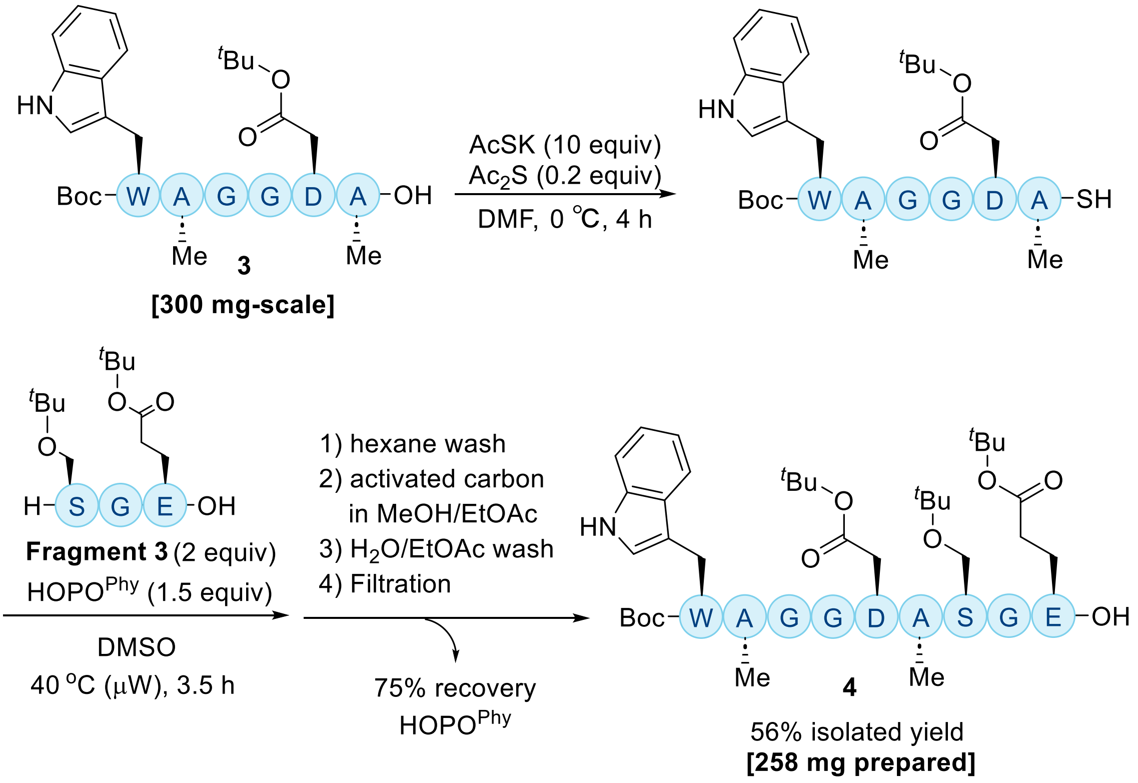

To a DMF solution (4.1 mL) of Boc-WAGGD(^{t}Bu)A-OH (**3**, 300 mg, 0.41 mmol) and potassium thioacetate (468 mg, 4.10 mmol, 10 equiv), diacetyl sulfide (8.6 μL, 0.082 mmol, 0.2 equiv) was added at 0 °C under an argon atmosphere. After stirring for 4 h at 0 °C, ethyl acetate, water, and 1 N HCl aq. were added. The aqueous layer was extracted with ethyl acetate (three times). The combined organic layers were washed with 1 N HCl aq., water, and saturated brine, and dried over Na_2SO_4. Volatiles were removed under reduced pressure and used for next reaction.

The crude H-S(^{t}Bu)GE(^{t}Bu)-OH (**Fragment 3**, 0.82 mmol, 2 equiv), HOPO^{Phy} (268 mg, 0.615 mmol, 1.5 equiv), and the crude Boc-WAGGD(^{t}Bu)A-OH (obtained as above) were dissolved in DMSO (10

mL) and the mixture was stirred at room temperature under air. After stirring at 40 °C for 3.5 h using a microwave apparatus, the mixture was transferred to another flask, rinsing with ethyl acetate and methanol. The solvents were removed under reduced pressure and remained DMSO was removed by lyophilization. To the resulting residue, hexane (50 mL) was added and the mixture was subjected to sonication. Precipitates were collected by filtration while washing with hexane. The filtrate was concentrated under reduced pressure and the residue was subjected to column chromatography (neutral silica gel, ethyl acetate/hexane = 20:80 → 70:30) to give recovered HOPO^{Phy} (202 mg, 75% recovery).

The filtered crude product **4** was mixed with methanol (50 mL) and ethyl acetate (20 mL), and after sonication to confirm complete dissolution, activated carbon (300 mg) was added. The mixture was stirred at 80 °C for 10 min. The mixture was then cooled to room temperature and filtered through Celite pad while washing with methanol and chloroform. Volatiles were removed under reduced pressure, then ethyl acetate was added to the residue, and applied to sonication. The product was filtered, rinsing while washing with water and ethyl acetate to obtain the target compound. The filtrate was concentrated under reduced pressure and filtered while washing with ethyl acetate and water to obtain further amount of target compound. The filtered compound was dissolved in methanol/chloroform, and the solvents were removed under reduced pressure. Then acetonitrile and water were added, then the mixture was subjected to lyophilization to give nonapeptide **4** as white amorphous solid (258 mg, 56% yield).

5-5. Procedure for global deprotection

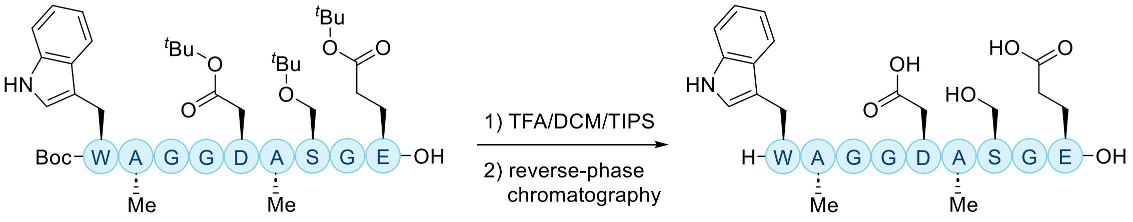

4
[243 mg-scale]

Delta-Sleep Inducing Peptide (DSIP)
62% isolated yield
[130 mg prepared]

To solution of nonapeptide **4** (243 mg, 0.218 mmol) and triisopropylsilane (TIPS, 250 μL) in dichloromethane (DCM, 7.5 mL), trifluoroacetic acid (TFA, 7.5 mL) was added at room temperature under an argon atmosphere. After stirring for 3 hours at room temperature, volatiles were removed under reduced pressure. The residue was dissolved in dichloromethane and volatiles were evaporated. This process was repeated for three times to eliminate volatile byproducts. The resulting residue was subjected to reversed-phase column chromatography (ODS, 0.1% TFA aq./acetonitrile = 98:2 → 50:50). Collected fractions were concentrated by lyophilization to afford **Delta-Sleep Inducing Peptide (DSIP)** as white solid (130.5 mg, 62%).

6. Reaction Profiling

A solution of Cbz-Phe-Val-SH (**1b**, 100 mg, 0.24 mmol), L-alanine (42.8 mg, 0.48 mmol, 2.0 equiv), and HOPO^{Phy} (157 mg, 0.36 mmol, 1.5 equiv) in DMSO (1.2 mL) and toluene (1.2 mL) was heated with stirring at 40 °C in a microwave device. At 10 min, 30 min, 1 h, 1.5 h, 3 h, 4.5 h, and 6 h, the test tube was removed from the microwave apparatus, and HPLC samples were prepared by mixing 12 μL of the reaction mixture and 68 μL of 1% TFA/DMSO. HPLC analysis was performed by method A. The peak area of each compound was compared with arbitrary unit (a.u.). The reaction profile was summarized in Figure 3-c in the main text.

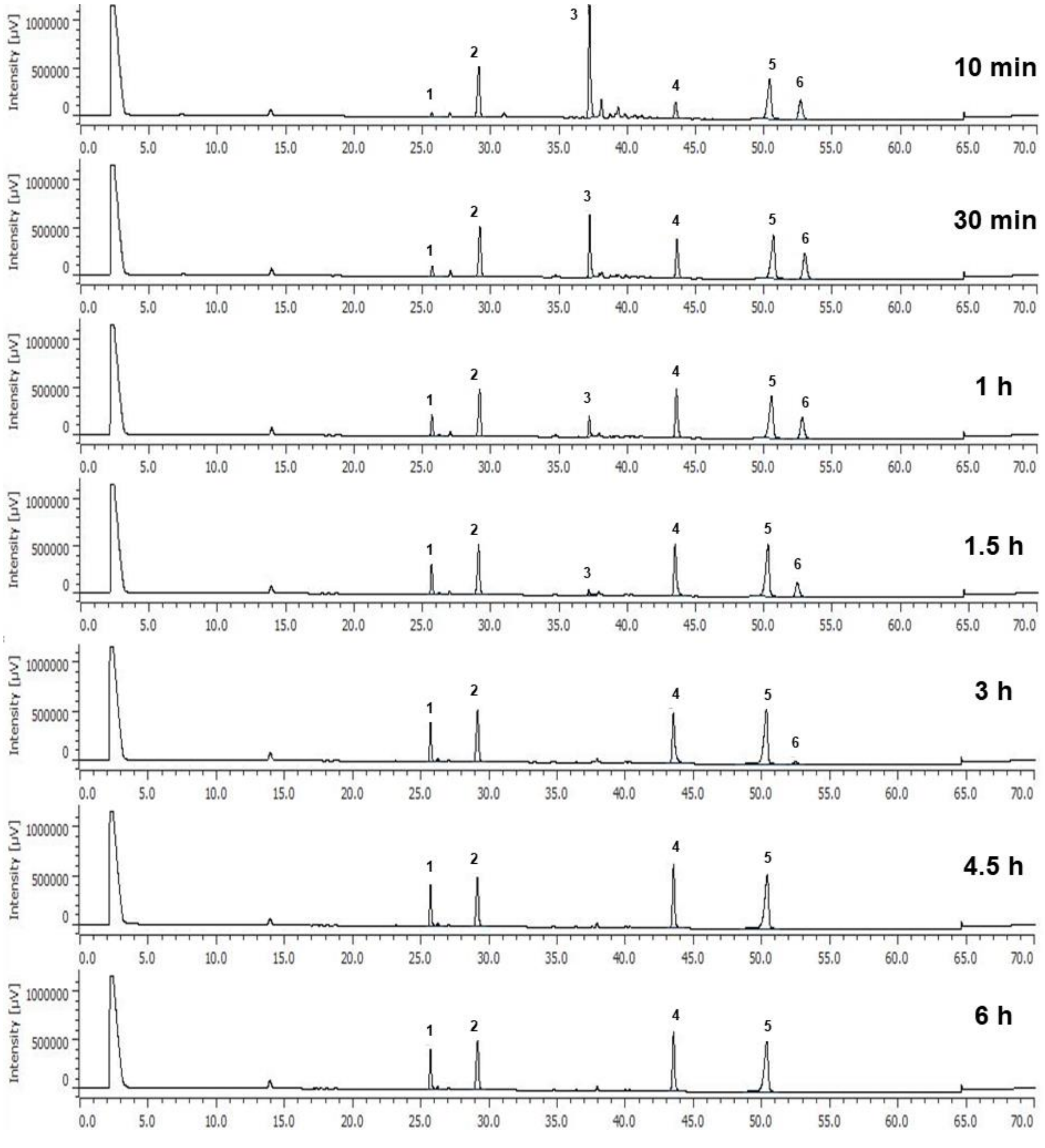

1: tripeptide **2ba**, 2: toluene, 3: disulfide **5**, 4: S_8, 5: HOPO^{Phy}, 6: active ester **6**

Figure S1. HPLC trace of the reaction profile

7. Oxygen Effects

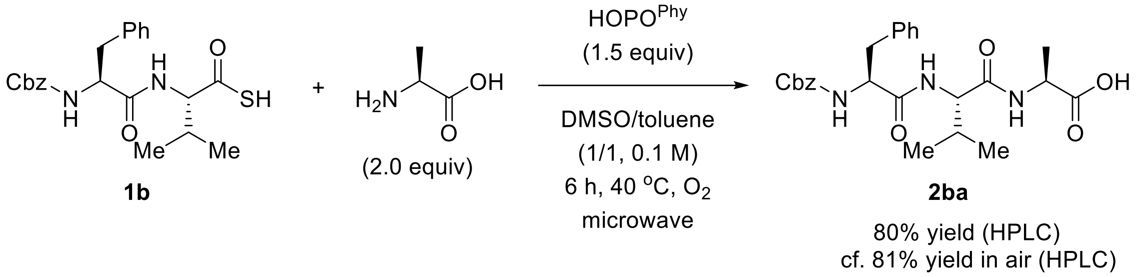

A solution of Cbz-Phe-Val-SH (**1b**, 100 mg, 0.24 mmol), L-alanine (42.8 mg, 0.48 mmol, 2.0 equiv), and HOPO^{Phy} (157 mg, 0.36 mmol, 1.5 equiv) in DMSO (1.2 mL) and toluene (1.2 mL) was heated with stirring at 40 °C in a microwave device under an oxygen atmosphere. At 10 min, 30 min, 1 h, 1.5 h, 3 h, 4.5 h, and 6 h, the test tube was removed from the microwave apparatus, and HPLC samples were prepared by mixing 12 μL of the reaction mixture and 68 μL of 1% TFA/DMSO. HPLC analysis was performed by method A. By comparison with the result under air, the acceleration effect of the reaction by oxygen was not observed.

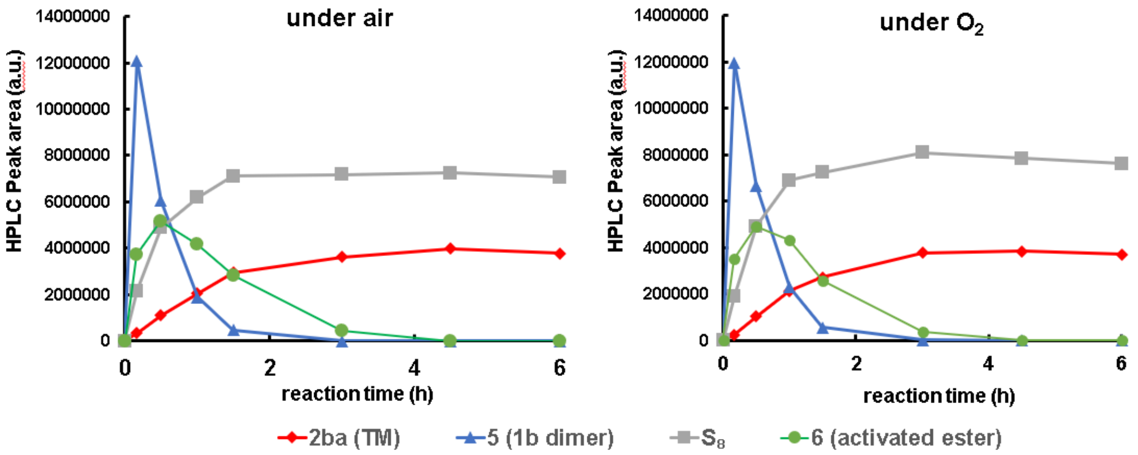

Figure S2. Oxygen effects

8. Sensitivity of PTC toward Hydrolysis

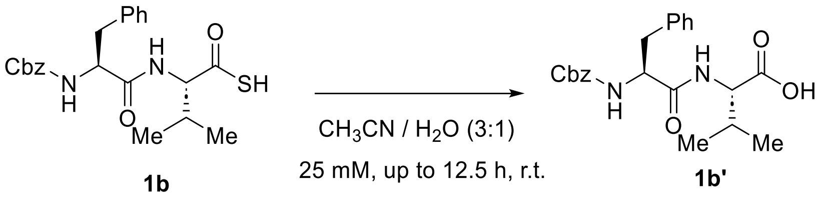

A solution of Cbz-Phe-Val-SH (**1b**, 10 mg, 0.048 mmol) in acetonitrile (0.72 mL) and water (0.24 mL) was stirring at room temperature under air. HPLC samples were prepared by mixing 20 µL of the reaction mixture and 80 µL of 25% water/acetonitrile. HPLC analysis was performed by method C. Although PTC is hydrolytically sensitive, it still contains enough lifetime for aqueous workup and the peptide coupling reaction. Some PTCs can be isolated by careful silica gel column purification (see Section 4-1).

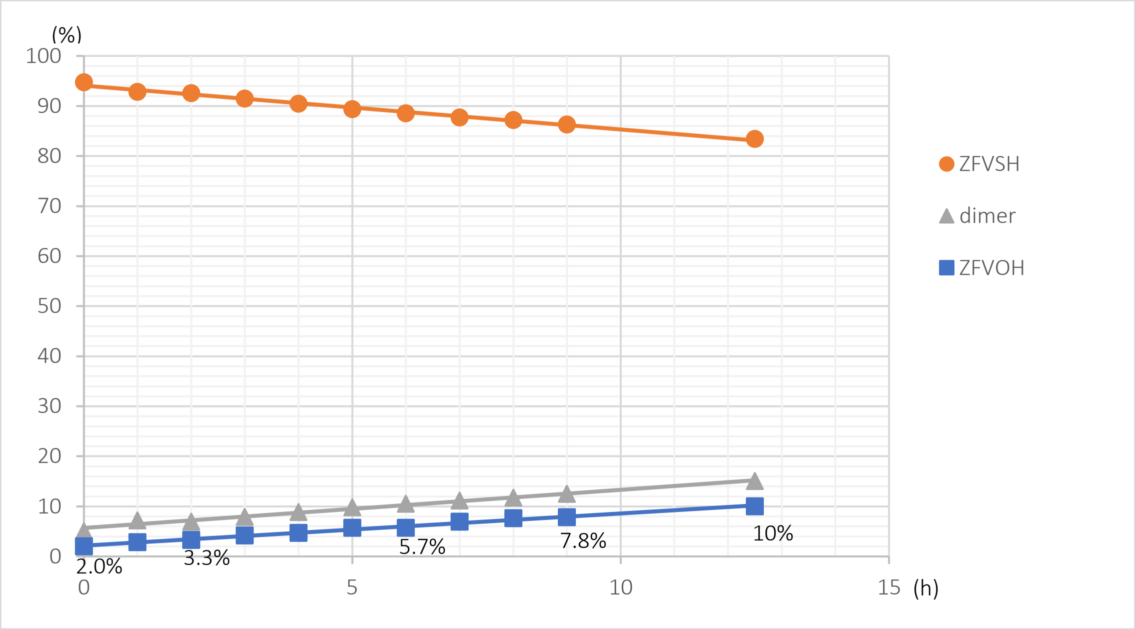

Figure S3. Stability evaluation of PTC in aqueous solvent

9. Preliminary Studies toward Application to Solid Phase Peptide Synthesis

We examined the solid-phase PTC-based N-to-C peptide synthesis using inverse 2-Cl trityl resin protocol.[4] The PTC synthesis and condensation with benzylamine, which has less solubility concern, were attempted as shown below. The desired amide was produced only in low yield (5–8%) and starting phenylalanine was majorly recovered after cleavage from the resin under acidic conditions. We speculate two main reasons for the low yield of the on-resin reaction; PTC synthesis was not sufficient and oxidative dimerization hardly proceeded. Therefore, the application of the current method to the solid-phase synthesis requires further intensive studies.

Table S3. Preliminary application to solid phase peptide synthesis

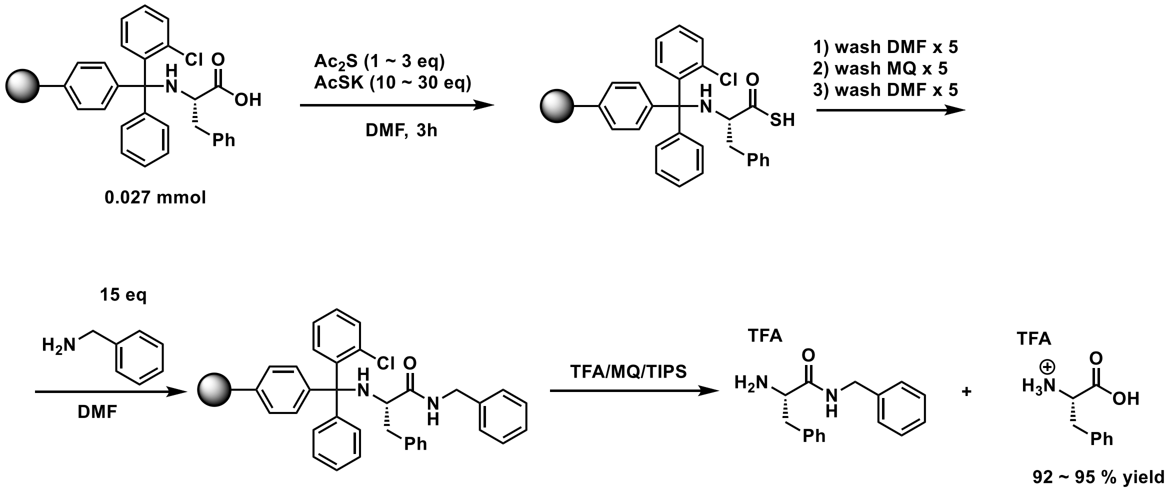

Entry	Ac$_2$S	AcSK	Yield
1	1	10	5
2	3	30	8

Yield was determined by LCMS.

10. Analytical Data

10-1 Peptide thiocarboxylic acid (PTC) 1

Cbz-Phe-Phe-SH (**1a**)

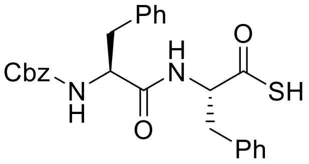

Isolated yield: 21%

^{1}H NMR (500 MHz, $CDCl_3$): δ 6.92-7.44 (m, 15H), 6.26-6.44 (m, 1H), 4.92-5.20 (m, 3H), 4.80-4.90 (m, 1H), 4.20-4.47 (m, 1H), 2.78-3.20 (m, 1H);

LRMS (ESI) m/z: [M+H]+ calcd. for $C_{26}H_{27}N_2O_4S$, 463.17; found; 463.29

LC-MS: t_R = 26.1 min, method E.

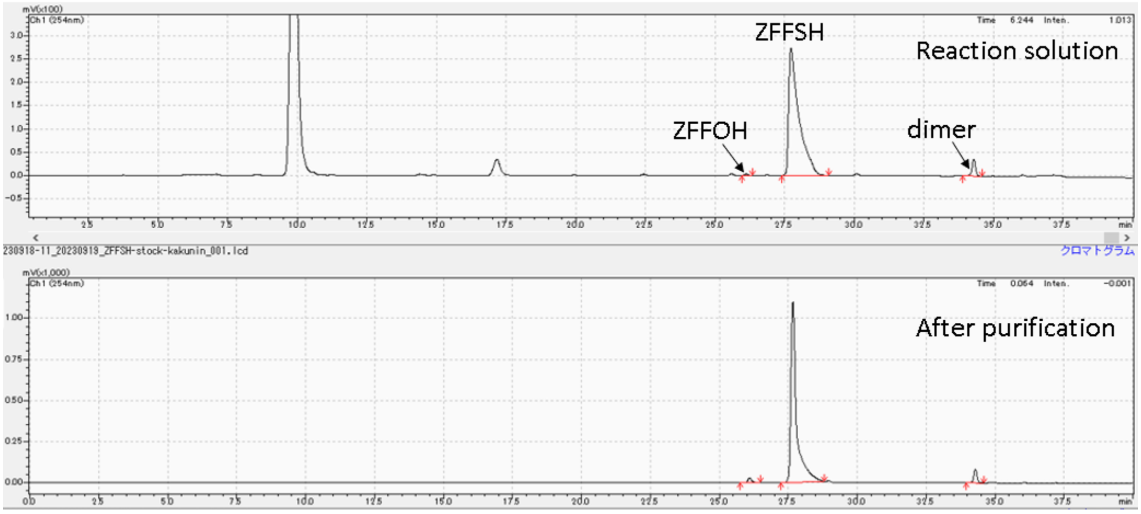

Cbz-Phe-Val-SH (**1b**)

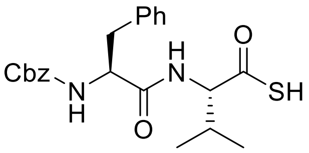

Isolated yield: 69%

^{1}H NMR (500 MHz, $CDCl_3$): δ 7.06-7.48 (m, 10H), 6.36-6.68 (br, 1H), 5.21-5.44 (br, 1H), 4.95-5.20 (m, 2H), 4.26-4.68 (m, 2H), 3.02-3.08 (m, 2H), 2.10-2.32 (m, 1H), 0.88 (d, J = 6.9 Hz, 3H), 0.79 (d, J = 6.3 Hz, 3H);

LRMS (ESI) m/z: [M+H]+ calcd. for $C_{22}H_{27}N_2O_4S$, 415.17; found; 415.15

LC-MS: t_R = 27.8 min, method F.

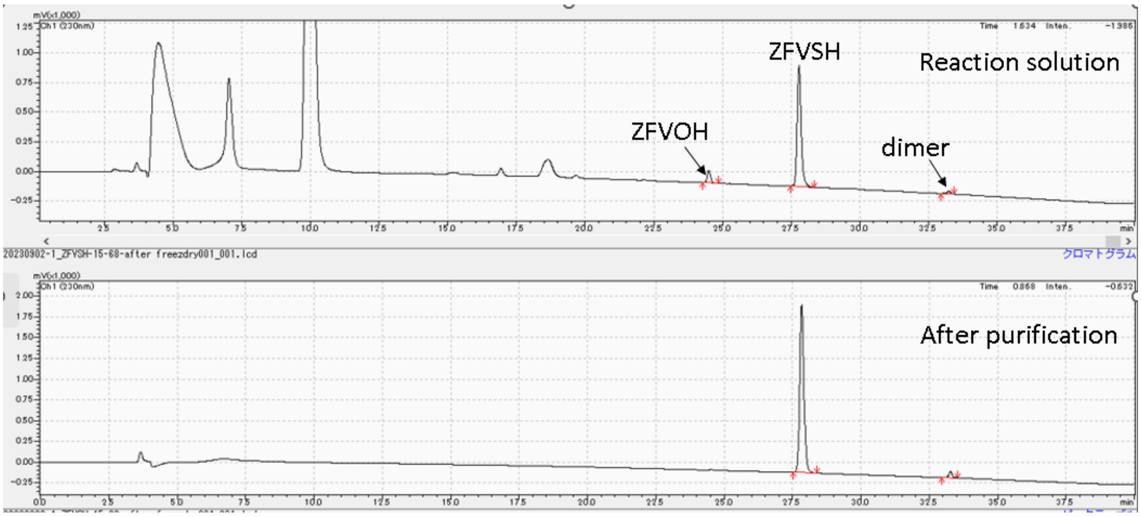

Fmoc-Phe-Val-SH (**1c**)

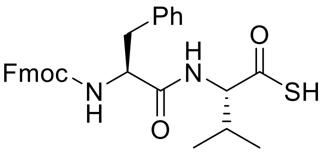

Isolated yield: 57%

^{1}H NMR (500 MHz, $CDCl_3$): δ 7.76 (d, J = 7.4 Hz, 2H), 7.52 (d, J = 7.4 Hz, 2H), 7.40 (t, J = 7.4 Hz, 2H), 7.14-7.36 (m, 7H), 6.22-6.56 (br, 1H), 5.17-5.40 (br, 1H), 4.26-4.60 (m, 4H), 4.20 (t, J = 6.9 Hz, 1H), 2.94-3.26 (m, 2H), 2.12-2.38 (m, 1H), 0.90 (d, J = 6.3 Hz, 2H), 0.80 (d, J = 6.9 Hz, 2H);

LRMS (ESI) m/z: [M+H]+ calcd. for $C_{29}H_{31}N_2O_4S$, 503.20; found 503.28;

LC-MS: t_R = 31.8 min, method E

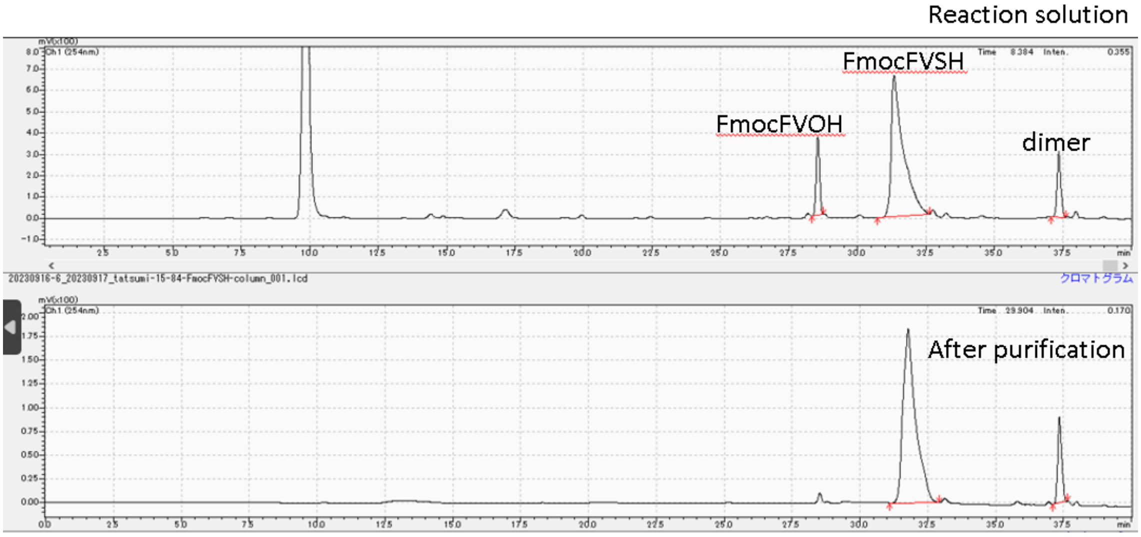

Boc-Phe-Val-SH (**1d**)

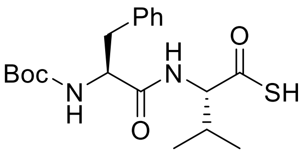

Isolated yield: 58%

^{1}H NMR (500 MHz, $CDCl_3$): δ 7.14-7.36 (m, 5H), 6.53-6.74 (br, 1H), 4.88-5.10 (br, 1H), 4.46-4.58 (m, 1H), 4.28-4.45 (m, 1H), 2.98-3.22 (m, 2H), 2.14-2.36 (m, 1H), 1.42 (s, 9H), 0.93 (d, J = 6.9 Hz, 3H), 0.84 (d, J = 6.9 Hz, 3H).

LRMS (ESI) m/z: [M+H]+ calcd. for $C_{19}H_{29}N_2O_4S$, 381.18; found 381.35;

LC-MS: t_R = 26.7 min, method E.

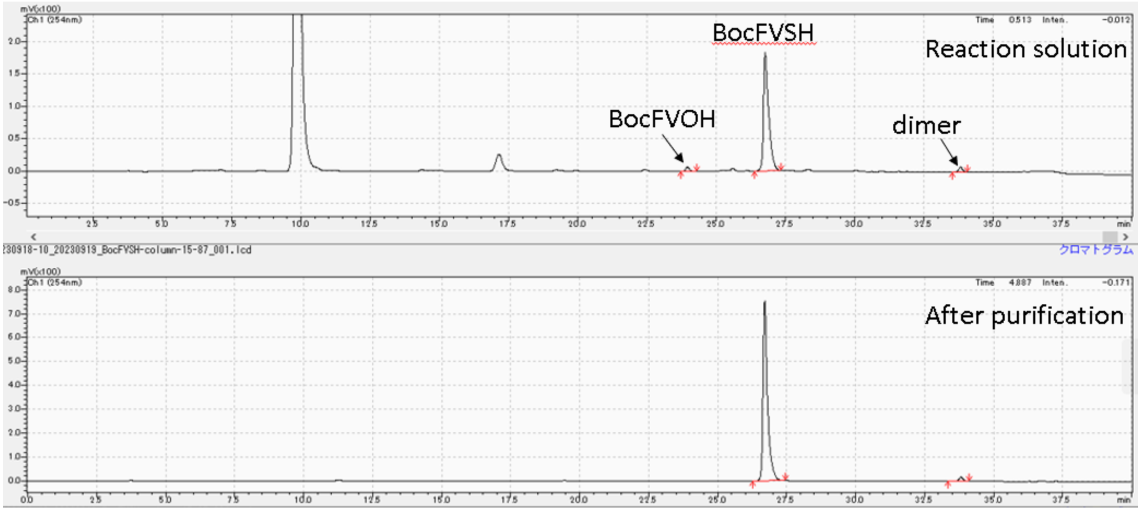

Cbz-Phe-His(Trt)-SH (**1e**)

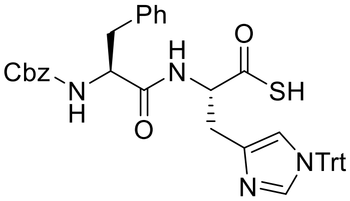

Isolated yield: 42%

^{1}H NMR (500 MHz, CD_3CN): δ 8.55 (s, 1H), 8.34 (s, 1H), 8.26-8.45 (m, 25H), 5.72-6.04 (br, 1H), 4.66-4.94 (m, 2H), 4.34-4.49 (br, 1H), 4.08-4.28 (br, 1H), 3.12-3.33 (m, 2H), 3.05 (dd, J = 4.6 Hz, 13.7 Hz, 1H), 2.85 (dd, J = 9.7 Hz, 13.7 Hz, 1H)

LRMS (ESI) m/z: [M+H]+ calcd. for $C_{42}H_{39}N_4O_4S$, 695.27; found 694.85;

LC-MS: t_R = 28.2 min, method E.

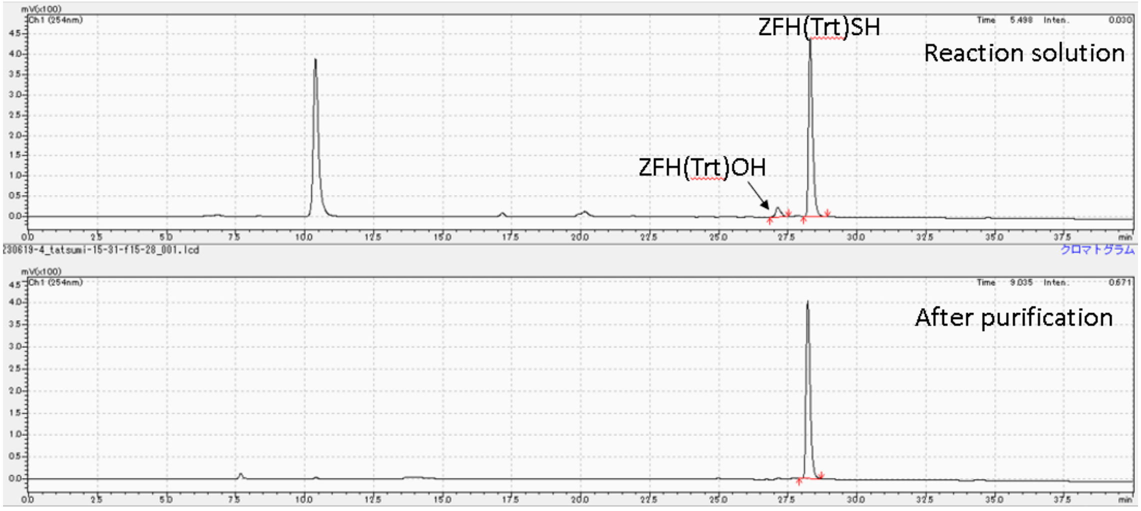

Cbz-Phe-Cys(Trt)-SH (**1f**)

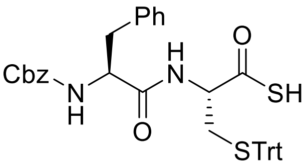

Isolated yield: 61%

^{1}H NMR (500 MHz, CD_3CN): δ 7.12-7.44 (m, 25H), 5.86-6.02 (m, 1H), 4.84-5.06 (m, 2H), 4.30-4.43 (m, 1H), 3.96-4.09 (m, 1H), 3.15 (dd, J = 5.2 Hz, 13.7 Hz, 1H), 2.84 (dd, J = 9.7 Hz, 13.7 Hz, 1H), 2.61 (dd, J = 8.6 Hz, 13.2 Hz, 1H), 2.53 (dd, J = 4.6 Hz, 13.2 Hz, 1H)

LRMS (ESI) m/z: [M+Na]+ calcd. for $C_{39}H_{36}N_2NaO_4S_2$, 683.20; found 683.25;

Analytical HPLC: t_R = 38.2 min, method A

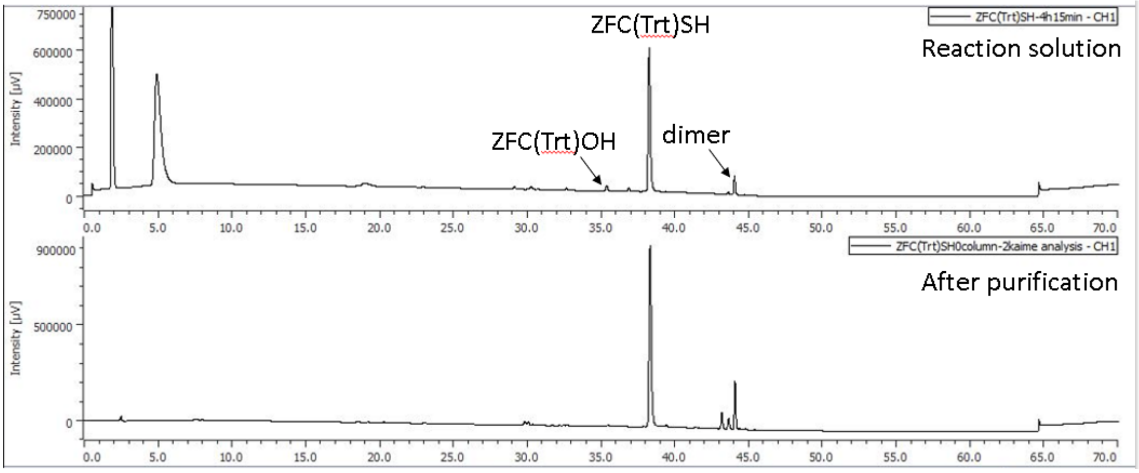

Cbz-Phe-Pro -SH (**1g**)

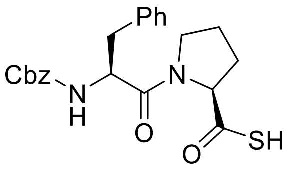

Isolated yield: 65%

^{1}H NMR (500 MHz, $CDCl_3$): δ 7.16-7.40 (m, 10H), 5.40-5.76 (m, 1H), 4.94-5.15 (m, 2H), 4.64-4.80 (m,2H), 3.56-3.76 (m, 1H), 3.06-3.22 (m, 1H), 2.83-3.05 (m, 1H), 1.80-2.22 (m, 4H)

LRMS (ESI) m/z: [M+H]+ calcd. for $C_{22}H_{25}N_2O_4S$, 413.15; found 413.36;

LC-MS: t_R = 26.0 min, method E.

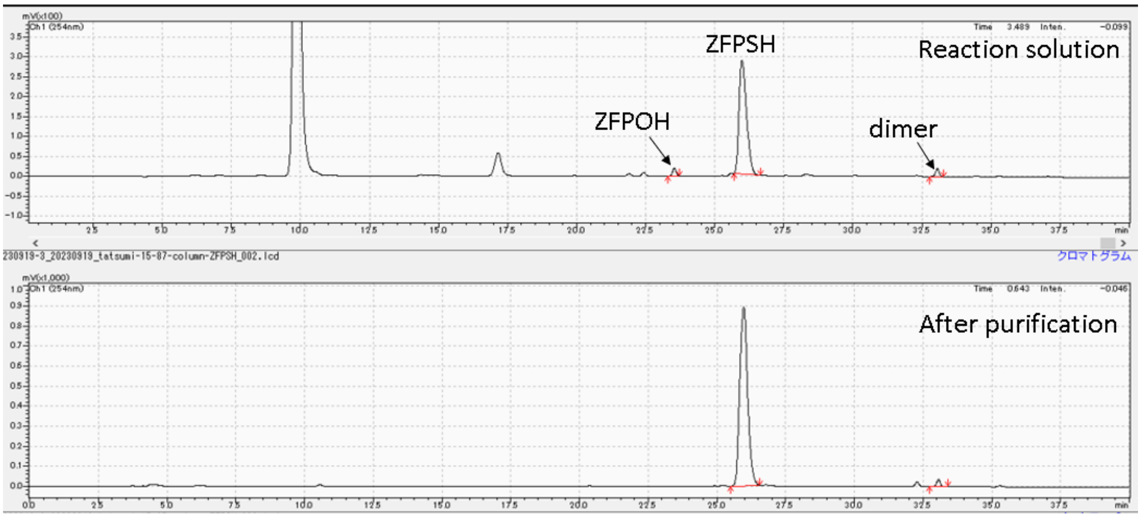

Boc-Trp-SH (**Boc-W-SH**)

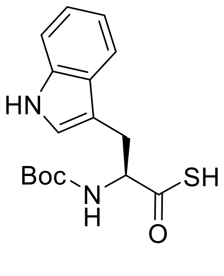

LRMS (ESI) m/z: [M+Na]+ calcd. for $C_{16}H_{21}N_2O_3S$, 321.13; found 321.17;

LC-MS: t_R = 21.0 min, method H.

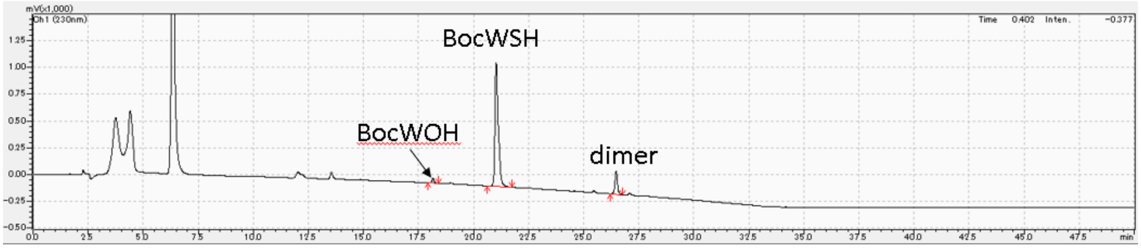

Boc-Trp-Ala-SH (**Boc-WA-SH**)

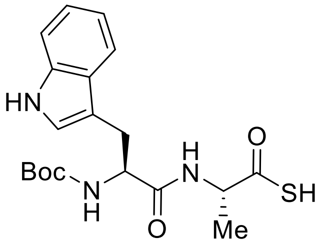

LRMS (ESI) m/z: [M+Na]+ calcd. for $C_{19}H_{25}N_3O_4NaS$,414.15; found 413.95;

LC-MS: t_R = 18.5 min, method G.

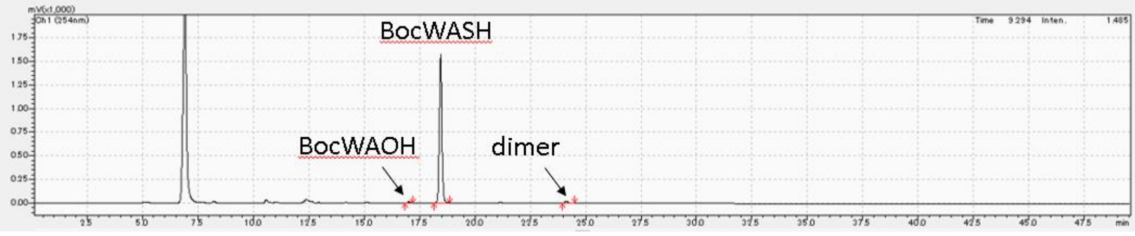

Fmoc-Gly-SH (**Fmoc-G-SH)**

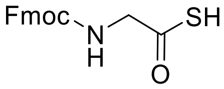

LRMS (ESI) m/z: [M+ Na]+ calcd. for $C_{17}H_{15}NNaO_3S$+, 336.07; found 335.90;

LC-MS: t_R = 20.4 min, method H.

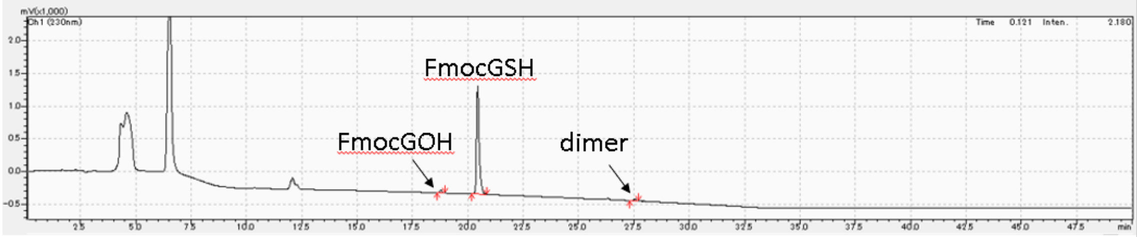

Fmoc-Gly-Asp(^{t}Bu)-SH (**Fmoc-GD(^{t}Bu)**)

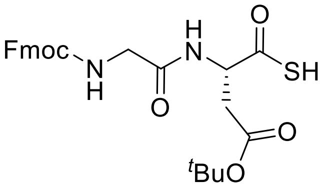

LRMS (ESI) m/z: [M+Na]+ calcd. for $C_{25}H_{28}N_2NaO_6S$, 507.16; found 507.00

LC-MS: t_R = 26.8 min, method E

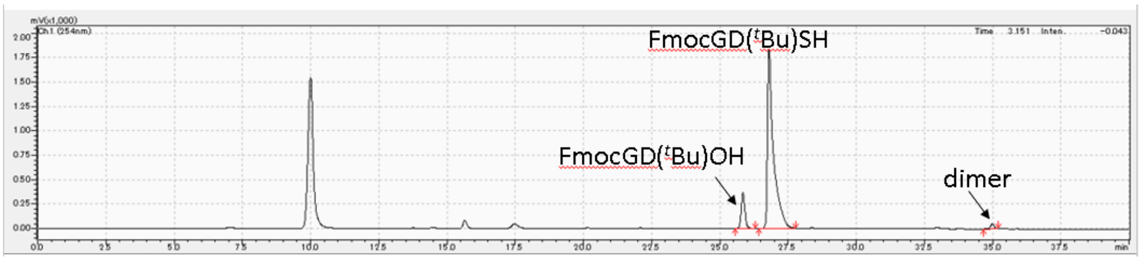

Fmoc-Ser(^{t}Bu)-SH (**Fmoc-S(^{t}Bu)-SH**)

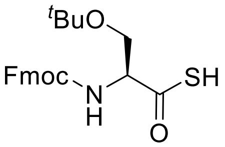

LRMS (ESI) m/z: [M+Na]+ calcd. for $C_{22}H_{25}NNaO_4S$, 422.14; found 421.90.

LC-MS: t_R = 25.6 min, method H

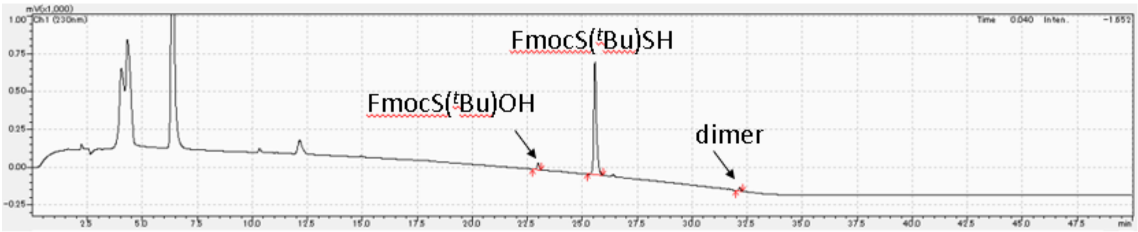

Fmoc-Ser(^{t}Bu)-Gly-SH(**Fmoc-S(^{t}Bu)G-SH**)

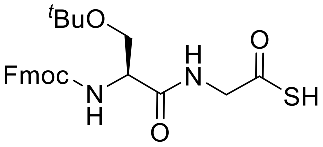

LRMS (ESI) m/z: [M+H]+ calcd. for $C_{24}H_{28}N_2NaO_5S$, 479.16; found 478.85;

LC-MS: t_R = 22.0 min, method H

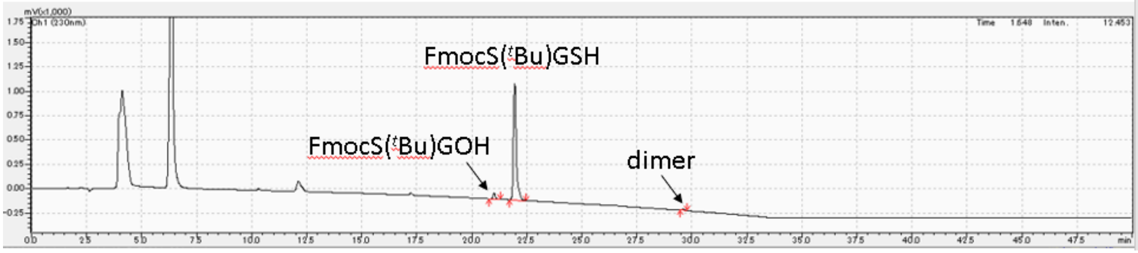

Boc-Trp-Ala-Gly-SH (**Boc-WAG-SH**)

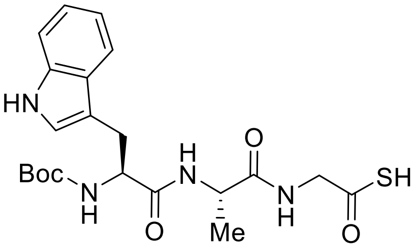

LRMS (ESI) m/z: [M+Na]+ calcd. for $C_{21}H_{28}N_4NaO_5S$, 471.17; found 470.90

LC-MS: t_R = 15.8 min, method H

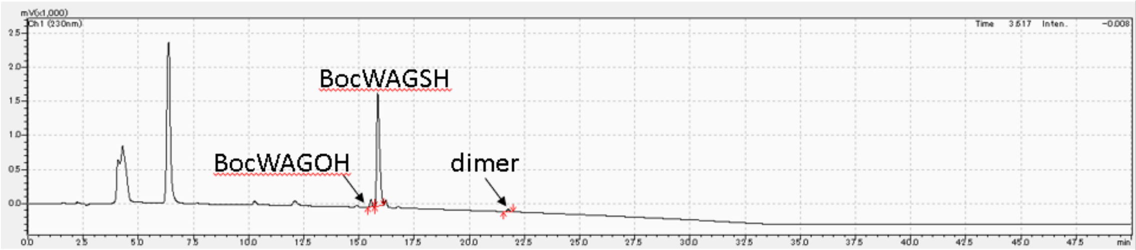

Boc-Trp-Ala-Gly-Gly-Asp(^{t}Bu)-Ala-OH (**Boc-WAGGD(tBu)A-SH**)

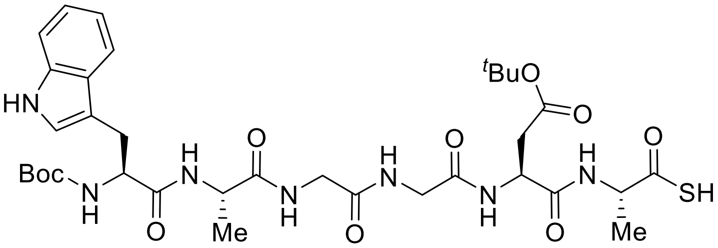

LRMS (ESI) m/z: [M+H]+ calcd. for $C_{34}H_{50}N_7O_{10}S$, 748.33; found 747.95

LC-MS: tR = 22.3 min, method E

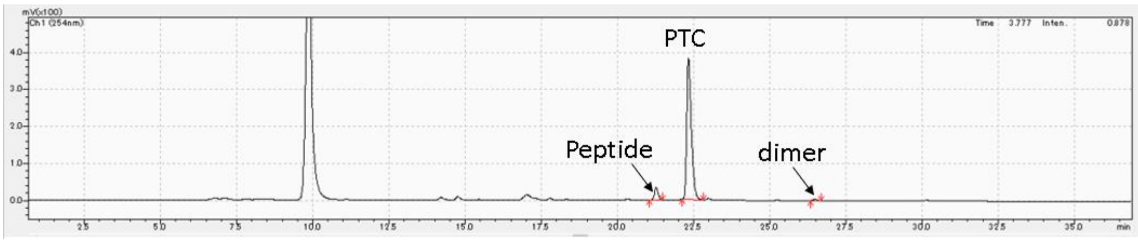

10-2 Oligopeptide products 2

We synthesized authentic epimer samples and assigned the HPLC peaks by comparing the retention times. The assignments of HPLC peaks other than the product and epimer were shown in Section 6.

Cbz-Phe-Val-Val-OH (**2bb**)

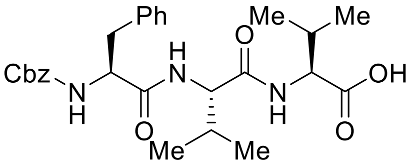

^{1}H NMR (500 MHz, CD_3OD): δ 8.24 (d, J = 7.8 Hz, 1H), 8.10 (d, J = 8.6 Hz, 1H), 7.10-7.30 (m, 11H), 5.00 (d, J = 12.6 Hz, 1H), 4.96 (d, J = 12.6 Hz, 1H), 4.40-4.60 (m, 1H), 4.18-4.39 (m, 2H), 3.10 (dd, J = 4.6 Hz, J = 13.7 Hz, 1H), 2.82 (dd, J = 10.3 Hz, J = 13.7 Hz, 1H), 1.96-2.22 (m, 2H), 0.82-1.04 (m, 12H);

^{13}C NMR (125.8 MHz, CD_3OD): δ174.5, 174.1, 173.7, 158.1, 138.5, 138.1, 130.4, 129.4, 129.3, 128.8, 128.6, 127.6, 67.5, 60.0, 59.0, 57.6, 39.1, 32.3, 31.7, 19.7, 19.6, 18.9, 18.5;

HRMS (ESI) *m/z*: $[M+Na]^+$ calcd. for $C_{27}H_{35}N_3NaO_6$, 520.2418; found 520.2428;

Analytical HPLC: t_R = 27.9 min (LLL isomer), 28.4 min (LDL isomer), method A.

Epimerization level of peptide coupling: <1% (using PTC **1b** with 5.3% epi. level)

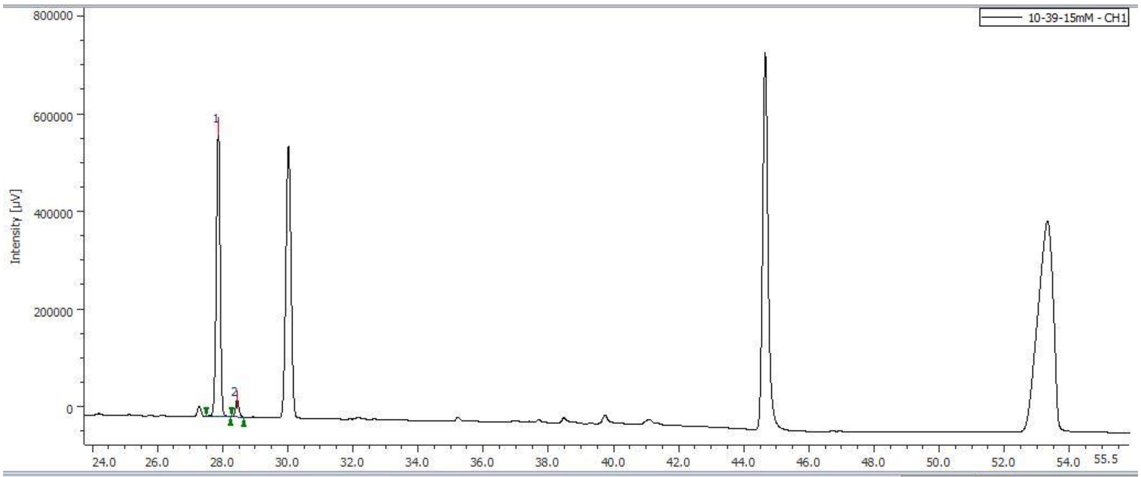

ピーク情報: 10-39-15mM

#	ピーク名	CH	tR	面積	高さ	面積%	高さ%	定量値	NTP	分離度	シンメトリー係数	警告
1	Unknown	1	27.875	4975652	594636	94.882	94.524	N/A	258734	2.659	0.883	
2	Unknown	1	28.442	268381	34448	5.118	5.476	N/A	298695	N/A	1.110	

Cbz-Phe-Val-Ala-OH (**2ba**)

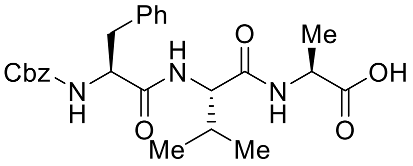

^{1}H NMR (391.8 MHz, DMSO-d_6): δ 8.30 (d, J = 6.7 Hz, 1H), 7.86 (d, J = 8.5 Hz, 1H), 7.54 (d, J = 9.0 Hz, 1H), 7.15-7.35 (m, 10H), 4.85-5.00 (m, 2H), 4.28-4.42 (m, 1H), 4.12-4.26 (m, 2H), 2.94-3.05 (m, 1H), 2.67-2.77 (m, 1H), 1.94-2.07 (m, 1H), 1.28 (d, J = 7.2 Hz, 3H), 0.89 (d, J = 6.7 Hz, 3H), 0.86 (d, J = 6.3 Hz, 3H);

^{13}C NMR (98.5 MHz, DMSO-d_6): δ 173.8, 171.2, 170.4, 155.8, 137.9, 136.9, 129.1, 128.2, 127.9, 127.6, 127.3, 126.2, 65.2, 57.2, 56.1, 47.5, 37.3, 30.9, 19.0, 17.8, 17.0;

HRMS (ESI) *m/z*: $[M+Na]^+$ calcd. for $C_{25}H_{31}N_3NaO_6$, 492.2105; found 492.2109;

Analytical HPLC: t_R = 25.7 min (LLL isomer), 26.2 min (LDL isomer), method A.

Epimerization level of peptide coupling: 2.1% (using PTC **1b** with 3.1% epi. level)

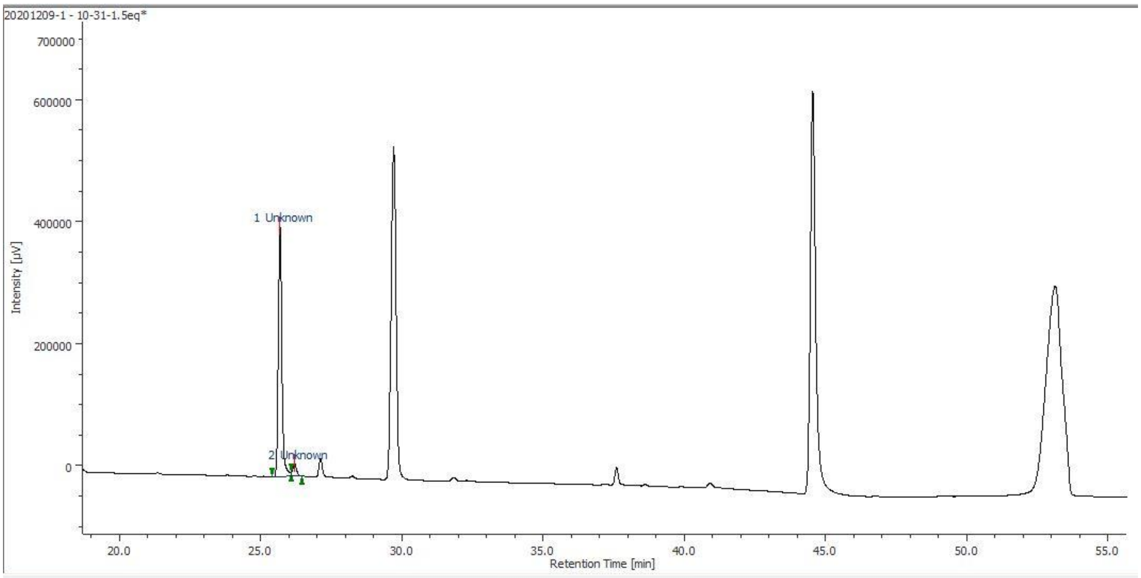

ピーク情報: 20201209-1 - 10-31-1.5eq*

#	ピーク名	tR	面積	高さ	面積%	高さ%	ピークスタート	ピークエンド	ベースラインスタート	ベースラインエンド	検出方法
1	Unknown	25.683	3595892	410307	94.907	94.982	25.417	26.083	25.417	26.458	マニュアル
2	Unknown	26.200	192960	21679	5.093	5.018	26.083	26.458	25.417	26.458	マニュアル

Cbz-Phe-Val-Gly-OH (**2bc**)

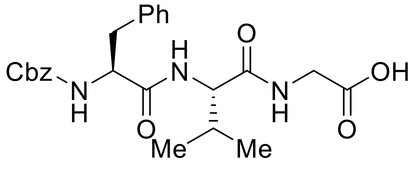

^{1}H NMR (391.8 MHz, CD_3OD): δ 8.08-8.14 (m, 1H), 7.97 (d, J = 8.5 Hz, 1H), 7.15-7.33 (m, 11H), 5.01 (s, 2H), 4.38-4.44 (m, 1H), 4.18-4.26 (m, 1H), 3.92-3.98 (m, 1H), 3.77-3.84 (m, 1H), 3.11 (dd, J = 5.4 Hz, J = 13.5 Hz, 1H), 2.85 (dd, J = 9.0 Hz, J = 13.5 Hz, 1H), 1.96-2.20 (m, 1H), 0.95 (d, J = 6.7 Hz, 3H), 0.92 (d, J = 6.7 Hz, 3H);

^{13}C NMR (125.8 MHz, CD_3OD): δ 174.0, 173.5, 172.5, 158.2, 138.5, 138.1, 130.4, 129.4, 128.9, 128.7, 127.7, 67.7, 59.9, 57.8, 41.8, 38.9, 32.1, 19.6, 18.4;

HRMS (ESI) *m/z*: $[M+Na]^+$ calcd. for $C_{24}H_{29}N_3NaO_6$, 478.1949; found 478.1955;

Analytical HPLC: t_R = 25.2 min (LLL isomer), 25.6 min (LDL isomer), method A.

Epimerization level of peptide coupling: 1.0% (using PTC **1b** with 5.3% epi. level)

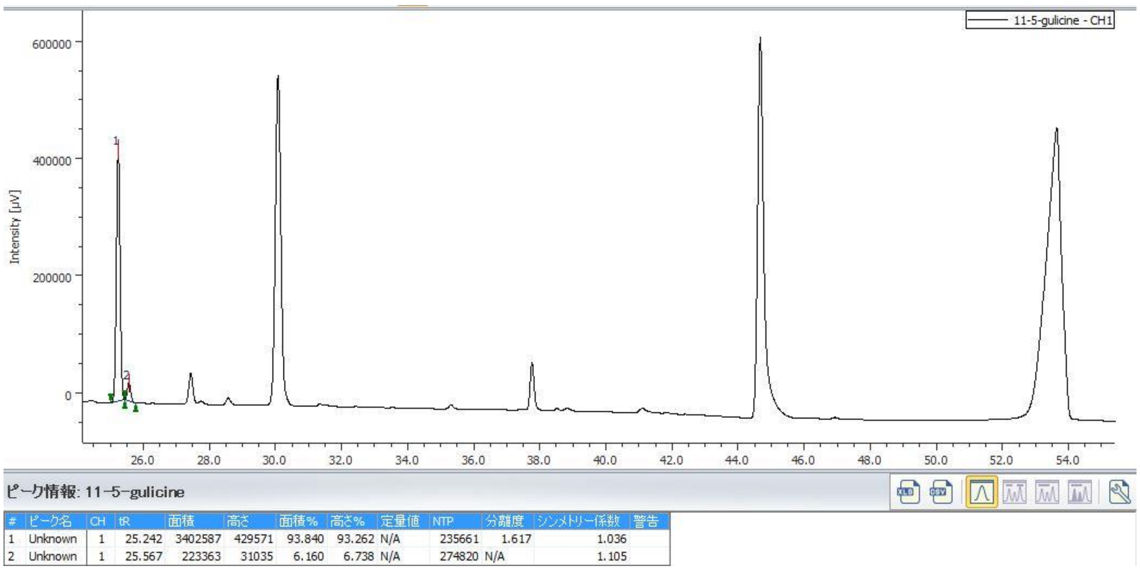

#	ピーク名	CH	tR	面積	高さ	面積%	高さ%	定量値	NTP	分離度	シンメトリー係数	警告
1	Unknown	1	25.242	3402587	429571	93.840	93.262	N/A	235661	1.617	1.036	
2	Unknown	1	25.567	223363	31035	6.160	6.738	N/A	274820	N/A	1.105	

Cbz-Phe-Val-Phe-OH (**2bd**)

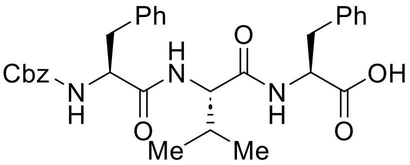

^{1}H NMR (391.8 MHz, DMSO-d_6): δ 8.28 (d, J = 7.6 Hz, 1H), 7.54 (d, J = 8.5 Hz, 1H), 7.15-7.32 (m, 16H), 4.85-5.00 (m, 2H), 4.38-4.46 (m, 1H), 4.29-4.33 (m, 1H), 4.18-4.24 (m, 1H), 3.06 (dd, J = 4.9 Hz, J = 13.9 Hz, 1H), 2.80-2.99 (m, 2H), 2.61-2.79 (m, 1H), 1.80-2.05 (m, 1H), 0.84 (d, J = 7.2 Hz, 3H), 0.81 (d, J = 6.7 Hz, 3H);

^{13}C NMR (98.5 MHz, DMSO-d_6): δ 182.1, 180.6, 180.1, 165.3, 147.5, 146.9, 146.4, 138.6, 138.4, 137.7, 137.6, 137.4, 137.0, 136.8, 136.7, 135.8, 135.6, 74.7, 66.8, 65.5, 62.8, 46.7, 40.3, 28.5, 27.3;

HRMS (ESI) m/z: $[M+Na]^+$ calcd. for $C_{31}H_{35}N_3NaO_6$, 568.2418; found 568.2427;

Analytical HPLC: t_R = 48.9 min (LLL isomer), 49.9 min (LDL isomer), method B.

Epimerization level of peptide coupling: 1.7% (using PTC **1b** with 5.3% epi. level)

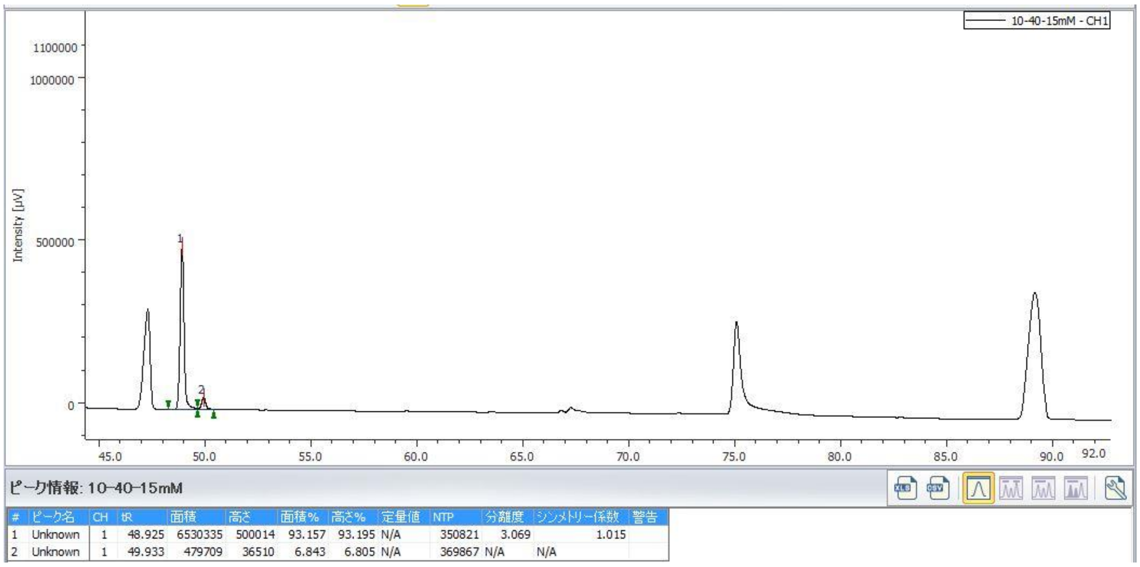

ピーク情報: 10-40-15mM

#	ピーク名	CH	tR	面積	高さ	面積%	高さ%	定量値	NTP	分離度	シンメトリー係数	警告
1	Unknown	1	48.925	6530335	500014	93.157	93.195	N/A	350821	3.069	1.015	
2	Unknown	1	49.933	479709	36510	6.843	6.805	N/A	369867	N/A	N/A	

Cbz-Phe-Val-Ile-OH (**2be**)

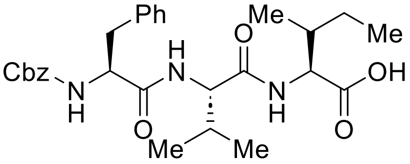

^{1}H NMR (391.8 MHz, CD_3OD): δ 7.16-7.31 (m, 10H), 4.94-5.01 (m, 2H), 4.36-4.49 (m, 2H), 4.23 (d, J = 7.6 Hz, 1H), 3.11 (dd, J = 4.5 Hz, J = 13.9 Hz, 1H), 2.81 (dd, J = 9.4 Hz, J = 13.9 Hz, 1H), 1.95-2.15 (m, 1H), 1.64-1.81 (m, 1H), 1.62 (t, J = 7.2 Hz, 2H), 0.89-0.96 (m, 12H);

^{13}C NMR (98.5 MHz, CD_3OD): δ 175.8, 174.0, 173.4, 158.2, 138.6, 138.2, 130.3, 129.4,129.4, 128.9, 128.7, 127.7, 67.5, 59.8, 57.7, 52.1, 41.6, 38.9, 32.4, 25.9, 23.4, 21.9, 19.7, 18.7;

HRMS (ESI) *m/z*: $[M+Na]^+$ calcd. for $C_{28}H_{37}N_3NaO_6$, 534.2575; found 534.2587;

Analytical HPLC: t_R = 48.3 min (LLL isomer), 49.2 min (LDL isomer), method B.

Epimerization level of peptide coupling: <1% (using PTC **1b** with 5.3% epi. level)

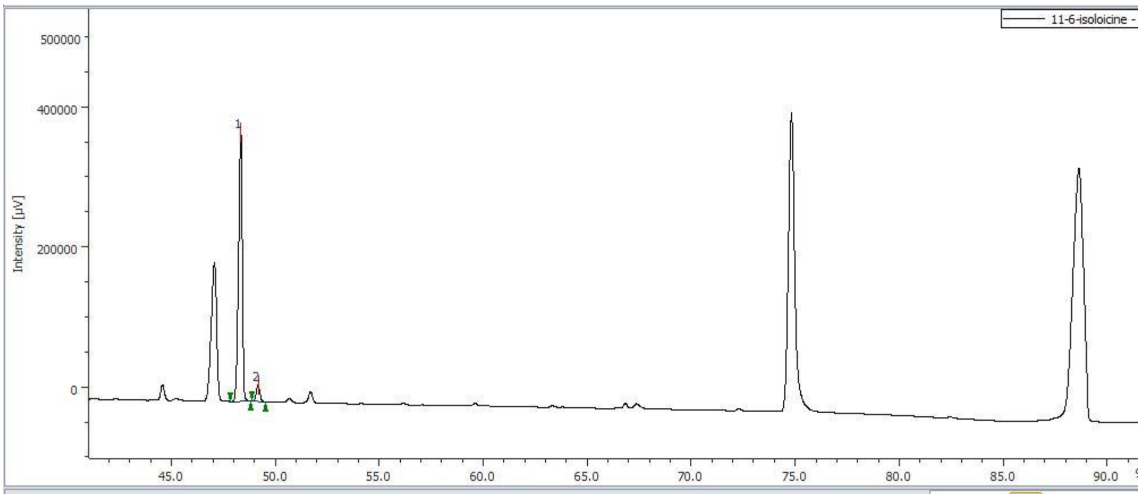

ピーク情報: 11−6−isoloicine

#	ピーク名	CH	tR	面積	高さ	面積%	高さ%	定量値	NTP	分離度	シンメトリー係数	警告
1	Unknown	1	48.333	4823983	383852	94.291	94.055	N/A	340959	2.575	0.840	
2	Unknown	1	49.167	292070	24263	5.709	5.945	N/A	382524	N/A	1.022	

Cbz-Phe-Val-Met-OH (**2bf**)

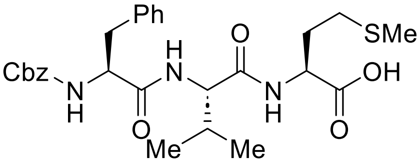

^{1}H NMR (391.8 MHz, CD_3OD): δ 7.19-7.30 (m, 11H), 5.00 (s, 2H), 4.37-4.52 (m, 2H), 4.10-4.24 (m, 1H), 3.06-3.19 (m, 1H), 2.83 (dd, J = 9.9 Hz, J = 13.9 Hz, 1H), 2.48-2.62 (m, 2H), 2.09-2.20 (m, 1H), 2.07 (s, 3H), 1.87-2.04 (m, 1H), 0.97 (d, J = 6.7 Hz, 3H), 0.94 (d, J = 7.2 Hz, 3H);

^{13}C NMR (125.8 MHz, CD_3OD): δ 174.7, 173.9, 173.3, 158.1, 138.5, 138.1, 130.3, 129.6, 129.4, 128.9, 128.7, 127.7, 67.7, 60.1, 57.7, 52.7, 39.0, 32.5, 32.2, 31.2, 19.6, 18.7, 15.3;

HRMS (ESI) *m/z*: $[M+Na]^+$ calcd. for $C_{27}H_{35}N_3NaO_6S$, 552.2139; found 552.2144;

Analytical HPLC: t_R = 28.0 min (LLL isomer), 28.4 min (LDL isomer), method A.

Epimerization level of peptide coupling: <1% (using PTC **1b** with 5.3% epi. level)

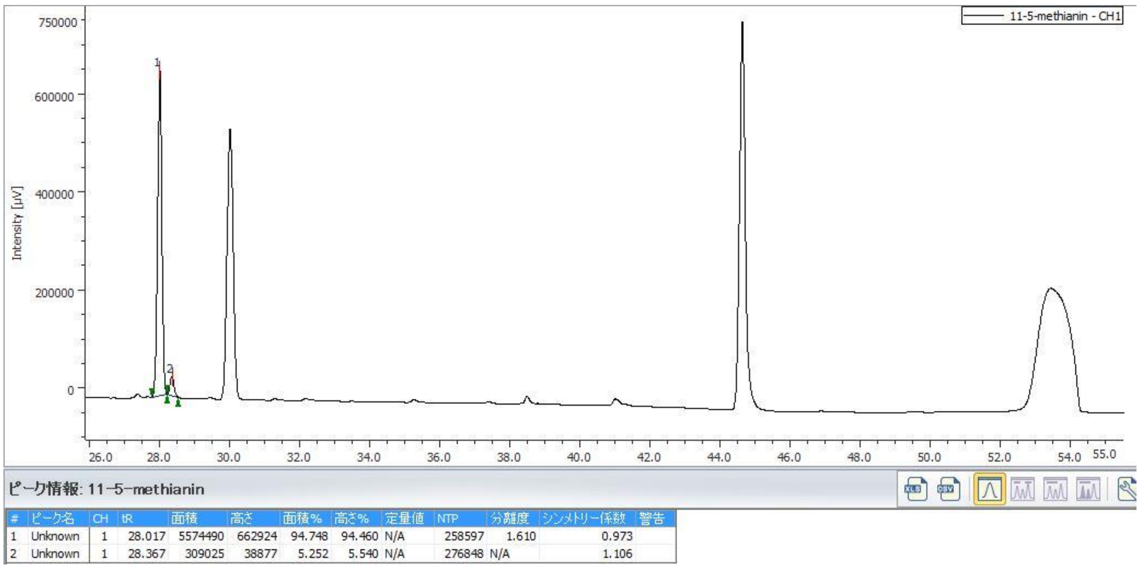

#	ピーク名	CH	tR	面積	高さ	面積%	高さ%	定量値	NTP	分離度	シンメトリー係数	警告
1	Unknown	1	28.017	5574490	662924	94.748	94.460	N/A	258597	1.610	0.973	
2	Unknown	1	28.367	309025	38877	5.252	5.540	N/A	276848	N/A	1.106	

Cbz-Phe-Val-Trp-OH (**2bg**)

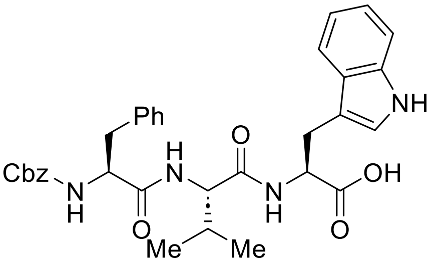

^{1}H NMR (500 MHz, DMSO-d_6): δ 10.83 (s, 1H), 8.28 (d, J = 7.4 Hz, 1H), 7.83 (d, J = 9.2 Hz, 1H), 7.52 (t, J = 8.6 Hz, 2H), 7.15-7.33 (m, 13H), 7.05 (t, J = 7.4 Hz, 1H), 6.98 (t, J = 7.4 Hz, 1H), 4.80-4.95 (m, 2H), 4.42-4.54 (m, 1H), 4.23-4.35 (m, 2H), 3.16 (dd, J = 5.7 Hz, J = 13.7 Hz, 1H), 3.05 (dd, J = 8.4 Hz, J = 13.7 Hz, 1H), 2.94 (dd, J = 3.4 Hz, J = 13.7 Hz, 1H), 2.69 (dd, J = 10.9 Hz, J = 13.7 Hz, 1H), 1.94-2.02 (m, 1H), 0.86 (d, J = 6.8 Hz, 3H), 0.82 (d, J = 6.9 Hz, 3H);

^{13}C NMR (125.8 MHz, DMSO-d_6): δ 173.3, 171.5, 171.0, 156.0, 138.2, 137.1, 136.2, 129.3, 128.4, 128.1, 127.8, 127.5, 127.3, 126.3, 123.7, 121.1, 118.5, 118.2, 111.5, 109.8, 65.3, 57.3, 56.1, 53.1, 37.4 31.1, 27.1, 19.3, 18.1;

HRMS (ESI) m/z: [M+Na]$^{+}$ calcd. for $C_{33}H_{36}N_4NaO_6$, 607.2527; found 607.2538;

Analytical HPLC: t_R = 48.9 min (LLL isomer), 49.6 min (LDL isomer), method B.

Epimerization level of peptide coupling: 1.5% (using PTC **1b** with 5.3% epi. level)

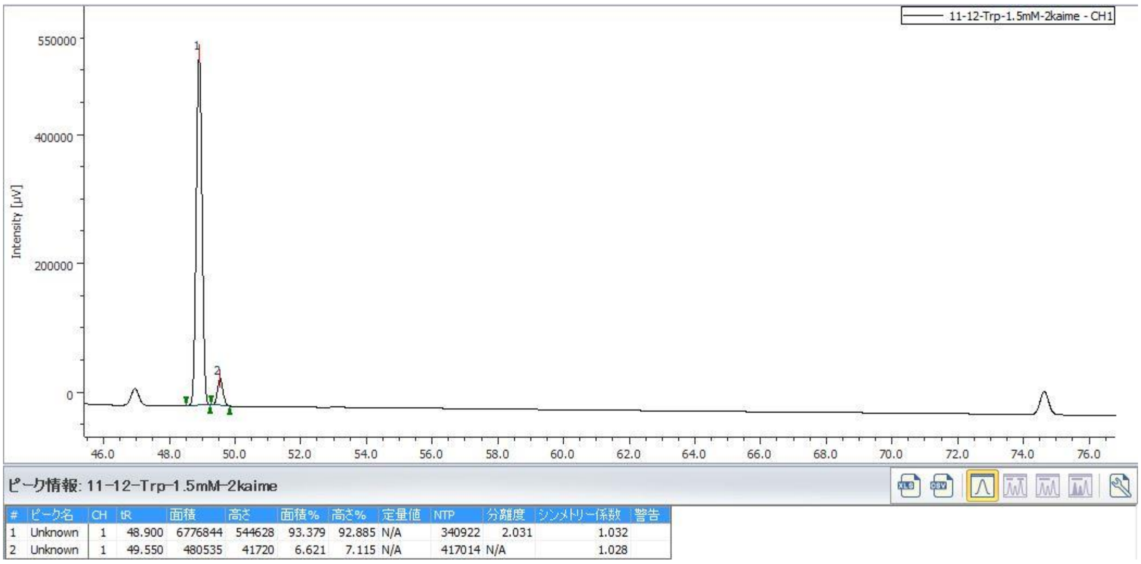

#	ピーク名	CH	tR	面積	高さ	面積%	高さ%	定量値	NTP	分離度	シンメトリー係数	警告
1	Unknown	1	48.900	6776844	544628	93.379	92.885	N/A	340922	2.031	1.032	
2	Unknown	1	49.550	480535	41720	6.621	7.115	N/A	417014	N/A	1.028	

Cbz-Phe-Val-Cys(Trt)-OH (**2bh**)

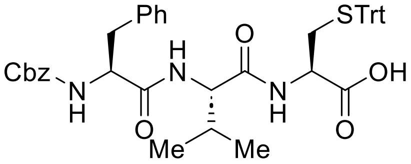

^{1}H NMR (391.8 MHz, DMSO-d_6): δ 7.94 (d, *J* = 9.4 Hz, 0.4H), 7.36 (m, 3H), 7.16-7.29 (m, 25H), 4.99 (s, 2H), 4.36-4.50 (m, 1H), 4.16-4.34 (m, 2H), 3.06-3.18 (m, 1H), 2.80 (dd, *J* = 9.9 Hz, *J* = 13.4 Hz, 1H), 2.59 (d, *J* = 6.3 Hz, 2H), 2.05 (m, 1H), 0.92 (d, *J* = 7.2 Hz, 3H), 0.90 (d, *J* = 6.7 Hz, 3H);
^{13}C NMR (98.5 MHz, DMSO-d_6): δ 171.6, 171.5, 170.9, 156.0, 147.9, 144.3, 138.2, 137.1, 129.4,129.3, 129.2, 128.4, 128.3, 128.2, 128.1, 127.9, 127.8, 127.7, 127.6, 127.5, 126.9, 126.8, 126.3, 66.3, 65.3, 57.3, 56.2, 51.5, 37.5, 33.0, 31.2, 19.2, 18.0;
HRMS (ESI) *m/z*: $[M+Na]^+$ calcd. for $C_{44}H_{45}N_3NaO_6S$, 766.2921; found 766.2924;
Analytical HPLC: t_R = 36.0 min (LLL isomer), 36.5 min (LDL isomer), method A.
Epimerization level of peptide coupling: <1% (using PTC **1b** with 5.3% epi. level)

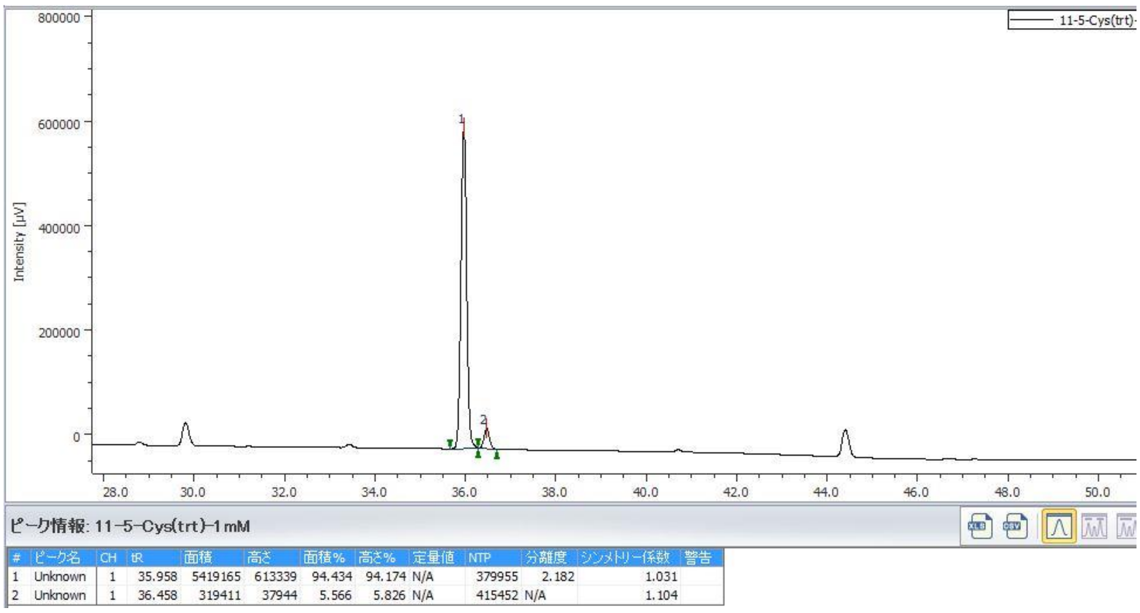

ピーク情報: 11-5-Cys(trt)-1 mM

#	ピーク名	CH	tR	面積	高さ	面積%	高さ%	定量値	NTP	分離度	シンメトリー係数	警告
1	Unknown	1	35.958	5419165	613339	94.434	94.174	N/A	379955	2.182	1.031	
2	Unknown	1	36.458	319411	37944	5.566	5.826	N/A	415452	N/A	1.104	

Cbz-Phe-Val-Ser-OH (**2bi**)

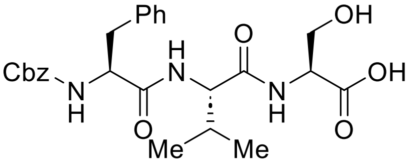

^{1}H NMR (391.8 MHz, DMSO-d_6): δ11.20 (s, 1H), 8.19 (d, J = 9.0 Hz, 1H), 7.91 (d, J = 9.4 Hz, 1H), 7.58 (d, J = 9.4 Hz, 1H), 7.17-7.33 (m, 10H), 4.94 (s, 2H), 4.30-4.40 (m, 3H), 3.03 (dd, J = 1.5 Hz, J = 13.9 Hz, 1H), 2.75 (dd, J = 11.2 Hz, J = 13.9 Hz, 1H), 1.90-2.10 (m, 1H), 0.88 (d, J = 6.7 Hz, 3H), 0.86 (d, J = 6.7 Hz, 3H);

^{13}C NMR (150.9 MHz, DMSO-d_6): δ 172.0, 171.5, 171.0, 156.0, 150.1, 138.3, 137.2, 129.4, 128.5, 128.2, 127.8, 127.5, 126.4, 65.3, 61.4, 57.3, 56.3, 54.9, 37.5, 31.2, 19.3, 18.1;

HRMS (ESI) *m/z*: $[M+Na]^+$ calcd. for $C_{25}H_{31}N_3NaO_7$, 508.2054; found 508.2061;

Analytical HPLC: t_R = 24.3 min (LLL isomer), 24.6 min (LDL isomer), method A.

Epimerization level of peptide coupling: <1% (using PTC **1b** with 5.3% epi. level)

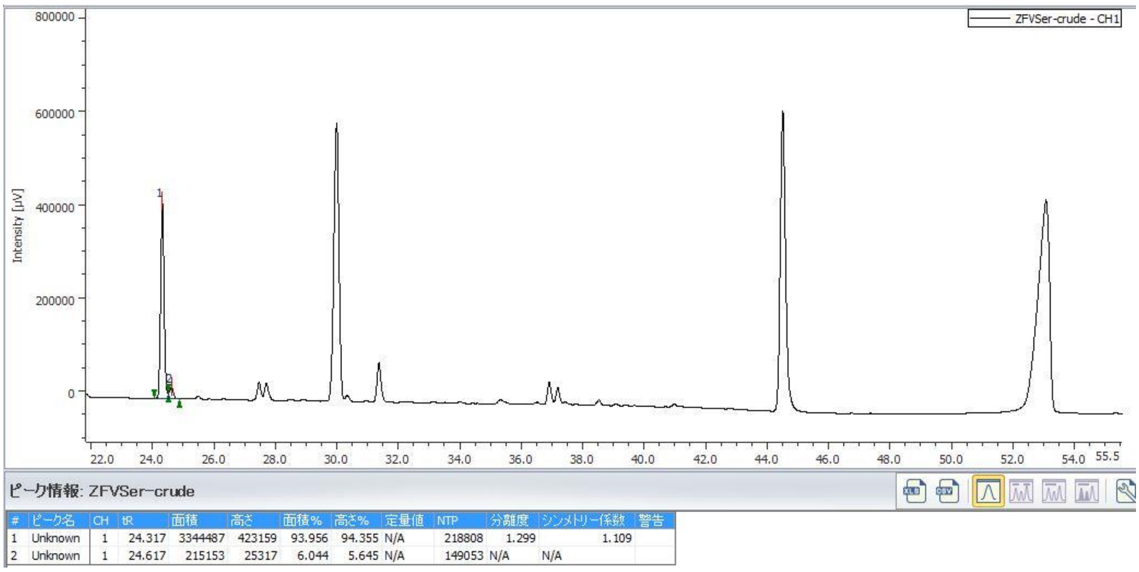

#	ピーク名	CH	tR	面積	高さ	面積%	高さ%	定量値	NTP	分離度	シンメトリー係数	警告
1	Unknown	1	24.317	3344487	423159	93.956	94.355	N/A	218808	1.299	1.109	
2	Unknown	1	24.617	215153	25317	6.044	5.645	N/A	149053	N/A	N/A	

Cbz-Phe-Val-Thr-OH (**2bj**)

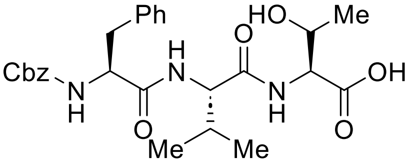

^{1}H NMR (391.8 MHz, CD_3OD): δ 8.05 (d, *J* = 8.1 Hz, 0.3H), 7.18-7.32 (m, 10H), 5.00 (s, 2H), 4.42-4.52 (m, 1H), 4.37-4.41 (m, 1H), 4.19-4.34 (m, 2H), 3.13 (dd, *J* = 4.5 Hz, *J* = 13.9 Hz, 1H), 2.82 (dd, *J* = 9.9 Hz, *J* = 13.9 Hz, 1H), 2.01-2.89 (m, 1H), 1.17 (d, *J* = 6.7 Hz, 3H), 0.81-1.05 (m, 6H);

^{13}C NMR (98.5 MHz, DMSO-d_6): δ 172.1, 171.6, 171.4, 156.0, 138.3, 137.1, 129.3, 128.4, 128.1, 127.8, 127.5, 126.4, 66.4, 65.3, 57.7, 57.6, 56.2, 37.4, 30.8, 20.5, 19.3, 18.1;

HRMS (ESI) *m/z*: $[M+Na]^+$ calcd. for $C_{26}H_{33}N_3NaO_7$, 522.2211; found 522.2219;

Analytical HPLC: t_R = 40.1 min (LLL isomer), 41.0 min (LDL isomer), method B.

Epimerization level of peptide coupling: <1% (using PTC **1b** with 6.8% epi. level)

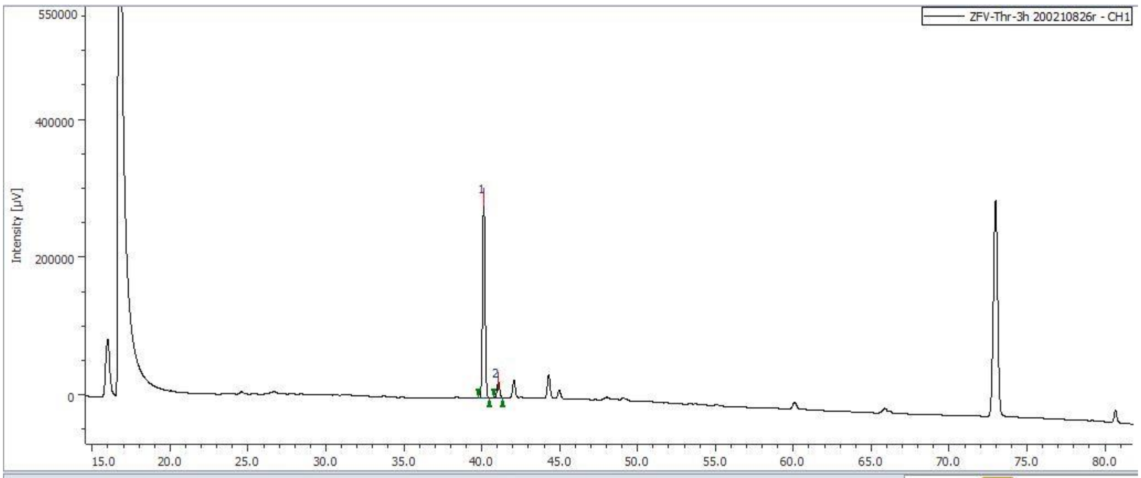

ピーク情報: ZFV-Thr-3h 200210826r

#	ピーク名	CH	tR	面積	高さ	面積%	高さ%	定量値	NTP	分離度	シンメトリー係数	警告
1	Unknown	1	40.100	3290618	291420	92.630	92.572	N/A	282238	3.109	1.062	
2	Unknown	1	41.033	261813	23385	7.370	7.428	N/A	299009	N/A	1.082	

Cbz-Phe-Val-Tyr-OH (**2bk**)

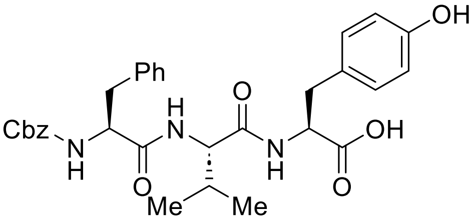

^{1}H NMR (500 MHz, CD$_3$OD): δ 8.21 (d, J = 8.0 Hz, 0.5H), 7.89 (d, J = 8.6 Hz, 0.7H), 7.16-7.30 (m, 11H), 7.03 (d, J = 8.6 Hz, 2H), 6.68 (d, J = 8.5 Hz, 2H), 4.98 (s, 2H), 4.53-4.62 (m, 1H), 4.38-4.46 (m, 1H), 4.15-4.26 (m, 1H), 2.98-3.13 (m, 2H), 2.88 (dd, J = 8.6 Hz, J = 14.3 Hz, 1H), 2.80 (dd, J = 10.8 Hz, J = 14.3 Hz, 1H), 1.92-2.06 (m, 1H), 0.90 (d, J = 6.9 Hz, 3H), 0.89 (d, J = 6.9 Hz, 3H);
^{13}C NMR (150.9 MHz, DMSO-d6): δ 173.1, 171.5, 171.1, 156.0, 138.4, 137.2, 130.1, 129.4, 128.4, 128.2, 127.8, 127.6, 127.5, 126.3, 115.1, 65.3, 57.4, 56.2, 54.0, 37.4, 36.1, 31.2, 19.3, 18.1HRMS (ESI) m/z: [M+Na]$^+$ calcd. for $C_{31}H_{35}N_3NaO_7$, 584.2367; found 584.2373;
Analytical HPLC: t_R = 43.9 min (LLL isomer), 44.3 min (LDL isomer), method B.
Epimerization level of peptide coupling: <1% (using PTC **1b** with 5.2% epi. level)

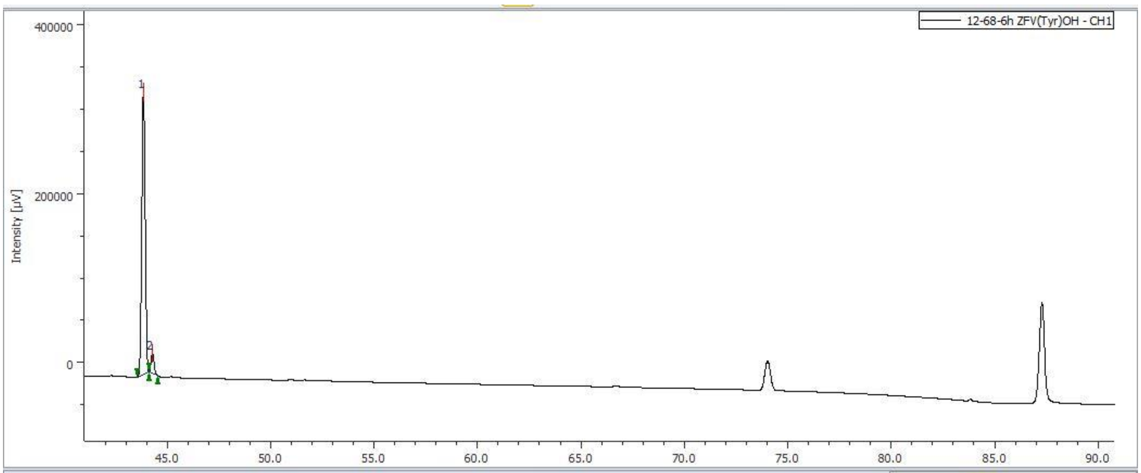

ピーク情報: 12-68-6h ZFV(Tyr)OH

#	ピーク名	CH	tR	面積	高さ	面積%	高さ%	定量値	NTP	分離度	シンメトリー係数	警告
1	Unknown	1	43.850	3826608	334100	94.130	93.378	N/A	330673	1.492	1.049	
2	Unknown	1	44.283	238642	23692	5.870	6.622	N/A	407590	N/A	1.131	

Cbz-Phe-Val-Asn-OH (**2bl**)

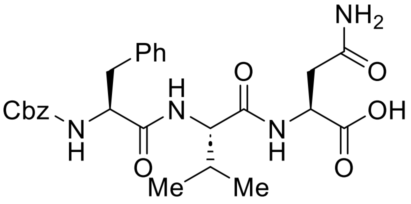

^{1}H NMR (600 MHz, DMSO-d_6): δ 8.21 (d, J = 7.2 Hz, 1H), 7.89 (d, J = 9.0 Hz, 1H), 7.52 (d, J = 8.4 Hz, 1H), 7.39 (s, 1H), 7.2-7.4 (m, 10H), 6.92 (s, 1H), 4.84-5.08 (m, 2H), 4.50-4.60 (m, 1H), 4.31-4.42 (m, 1H), 4.10-4.30 (m, 1H), 3.01 (d, J = 10.8 Hz, 1H), 2.73 (t, J = 10.8 Hz, 1H), 2.56 (dd, J = 6.0 Hz, 15.6 Hz, 1H), 2.46 (dd, J = 6.6 Hz, 15.6 Hz, 1H), 1.97-2.05 (m, 1H), 0.87 (d, J = 7.2 Hz, 3H), 0.84 (d, J = 6.6 Hz, 3H);

^{13}C NMR (98.5 MHz, CD_3OD): δ 182.3, 181.1, 180.9, 180.2, 165.5, 147.8, 146.6, 138.8, 137.9, 137.6, 137.3, 137.0, 135.8, 74.8, 66.8, 65.7, 58.4, 46.9, 46.2, 40.6, 28.7, 27.4;

HRMS (ESI) m/z: $[M+Na]^+$ calcd. for $C_{26}H_{32}N_4NaO_7$, 535.2163; found 535.2172;

Analytical HPLC: t_R = 37.4 min (LLL isomer), 37.7 min (LDL isomer), method B.

Epimerization level of peptide coupling: <1% (using PTC **1b** with 6.8% epi. level)

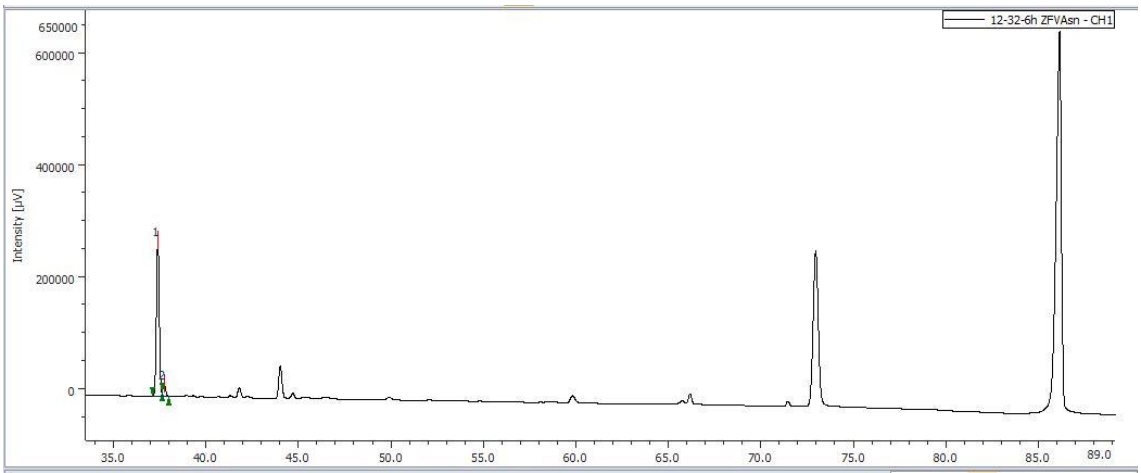

ピーク情報: 12-32-6h ZFVAsn

#	ピーク名	CH	tR	面積	高さ	面積%	高さ%	定量値	NTP	分離度	シンメトリー係数	警告
1	Unknown	1	37.400	2998657	279182	92.686	92.476	N/A	275667	1.204	0.999	
2	Unknown	1	37.742	236621	22716	7.314	7.524	N/A	282035	N/A	N/A	

Cbz-Phe-Val-Asp-OH (**2bm**)

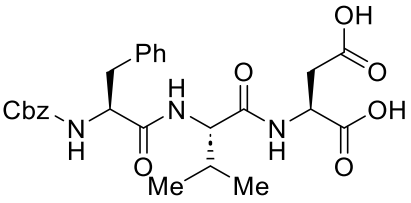

^{1}H NMR (391.8 MHz, CD_3OD): δ 8.28 (d, J = 7.6 Hz, 1H), 7.91 (d, J = 9.0 Hz, 1H), 7.53 (d, J = 9.0 Hz, 1H), 7.17-7.33 (m, 10H), 4.85-4.99 (m, 2H), 4.48-4.53 (m, H), 4.30-4.40 (m, 1H), 4.18-4.24 (m, 1H), 2.94-3.02 (m, 1H), 2.62-2.82 (m, 2H), 2.53-2.61 (m, 1H), 1.86-2.10 (m, 1H), 0.74-0.94 (m, 6H); ^{13}C NMR (98.5 MHz, DMSO-d_6): δ 172.4, 171.8, 171.6, 170.7, 156.0, 138.2, 137.1, 129.3, 128.4, 128.1, 127.8, 127.5, 126.3, 65.3, 57.3, 56.2, 48.7, 37.4, 36.2, 31.1, 19.2, 17.9;

HRMS (ESI) *m/z*: $[M+Na]^+$ calcd. for $C_{26}H_{31}N_3NaO_8$, 536.2003; found 536.2014;

Analytical HPLC: t_R = 39.4 min (LLL isomer), 39.8 min (LDL isomer), method B.

Epimerization level of peptide coupling: <1% (using PTC **1b** with 6.8% epi. level)

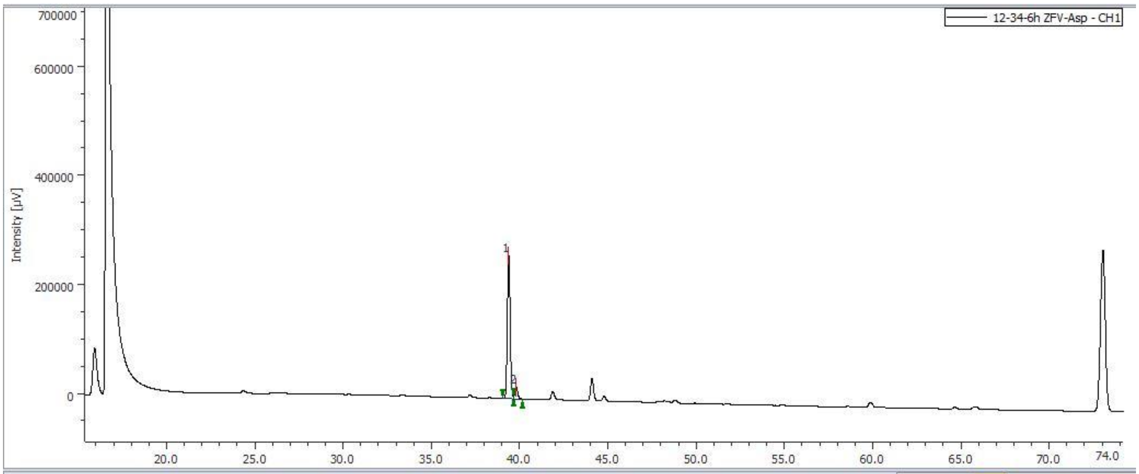

ピーク情報: 12-34-6h ZFV-Asp

#	ピーク名	CH	tR	面積	高さ	面積%	高さ%	定量値	NTP	分離度	シンメトリー係数	警告
1	Unknown	1	39.358	2950806	261684	92.558	92.391	N/A	271723	1.434	1.082	
2	Unknown	1	39.783	237253	21550	7.442	7.609	N/A	296583	N/A	N/A	

Cbz-Phe-Val-Lys(Boc)-OH (**2bn**)

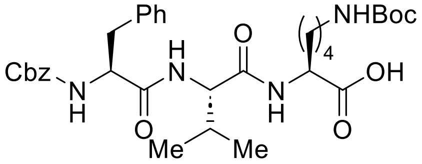

^{1}H NMR (600 MHz, DMSO-d_6): δ 8.16 (d, J = 7.2 Hz, 1H), 7.86 (d, J = 9.0 Hz, 1H), 7.53 (d, J = 8.4 Hz, 1H), 7.39 (s, 1H), 7.18-7.33 (m, 10H), 6.73-6.80 (m, 1H), 4.88-5.00 (m, 2H), 4.30-4.36 (m, 1H), 4.23-4.28 (m, 1H), 4.10-4.14 (m, 1H), 2.98 (dd, J = 3.6, 13.8 Hz, 1H), 2.80-2.90 (m, 2H), 2.72 (dd, J = 10.8, 13.8 Hz, 1H), 1.90-2.05 (m, 1H), 1.65-1.75 (m, 1H), 1.55-1.64 (m, 1H), 1.43 (s, 9H), 1.20-1.48 (m, 2H), 0.88 (d, J = 6.8 Hz, 3H), 0.85 (dd, J = 6.8 Hz, 3H);

^{13}C NMR (150.9 MHz, DMSO-d_6): δ 173.6, 171.5, 171.0, 156.0, 155.7, 138.3, 137.2, 129.4, 128.4, 128.2, 127.8, 127.5, 126.4, 77.5, 65.3, 57.3, 56.2, 52.2, 37.5, 31.2, 30.8, 29.3, 28.4, 22.9, 19.3, 18.1;

HRMS (ESI) *m/z*: $[M+Na]^+$ calcd. for $C_{33}H_{46}N_4NaO_8$, 649.3208; found 649.3213;

Analytical HPLC: t_R = 49.6 min (LLL isomer), 50.2 min (LDL isomer), method B.

Epimerization level of peptide coupling: 1.8% (using PTC **1b** with 5.3% epi. level)

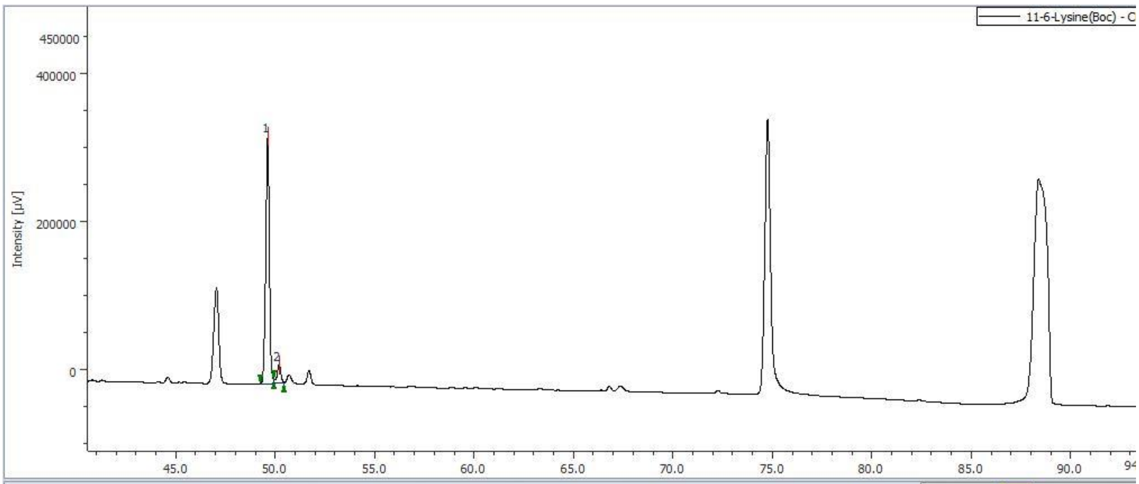

ピーク情報: 11-6-Lysine(Boc)

#	ピーク名	CH	tR	面積	高さ	面積%	高さ%	定量値	NTP	分離度	シンメトリー係数	警告
1	Unknown	1	49.625	4236312	333481	93.106	93.347	N/A	367107	1.725	0.914	
2	Unknown	1	50.192	313698	23766	6.894	6.653	N/A	367198	N/A	N/A	

Cbz-Phe-Val-Arg(Pbf)-OH (**2bo**)

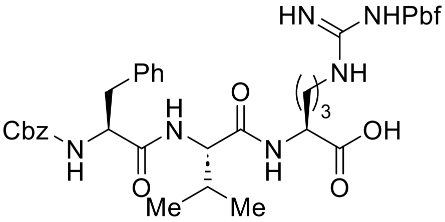

^{1}H NMR (600 MHz, DMSO-d_6): δ 8.20 (d, J = 7.2 Hz, 1H), 7.88 (d, J = 9.0 Hz, 1H), 7.52 (d, J = 8.4 Hz, 1H), 7.17-7.33 (m, 10H), 6.60-6.80 (br, 0.4H), 6.30-6.58 (br, 1H), 4.88-5.00 (m, 2H), 4.30-4.40 (m, 1H), 4.21-4.28 (m, 1H), 4.10-4.16 (m, 1H), 3.02-3.06 (m, 2H), 2.98 (dd, J = 3.6 Hz, J = 10.8 Hz, 1H), 2.94 (s, 3H), 2.72 (dd, J = 10.8, 13.8 Hz, 1H), 2.47 (s, 3H), 2.42 (s, 3H), 1.99 (s, 3H), 1.92-1.98 (m, 1H), 1.66-1.74 (m, 1H), 1.50-1.60 (m, 1H), 1.40-1.49 (m, 2H), 1.40 (s, 3H), 0.88 (d, J = 6.7 Hz, 3H), 0.85 (dd, J = 6.7 Hz, 3H);

^{13}C NMR (150.9 MHz, DMSO-d_6): δ 173.5, 171.5, 171.0, 157.6, 156.3, 156.0, 138.3, 137.4, 137.2, 131.6, 129.4, 128.4, 128.2, 127.8, 127.5, 126.4, 124.5, 116.4, 86.5, 70.0, 65.3, 57.4, 56.2, 51.9, 42.6, 37.5, 31.1, 28.5, 19.3, 19.1, 18.2, 17.8, 12.5;

HRMS (ESI) m/z: [M+Na]$^+$ calcd. for $C_{41}H_{54}N_6NaO_9S$, 829.3565; found 829.3561;

Analytical HPLC: t_R = 52.0 min (LLL isomer), 52.5 min (LDL isomer), method B.

Epimerization level of peptide coupling: 1.3% (using PTC **1b** with 5.3% epi. level)

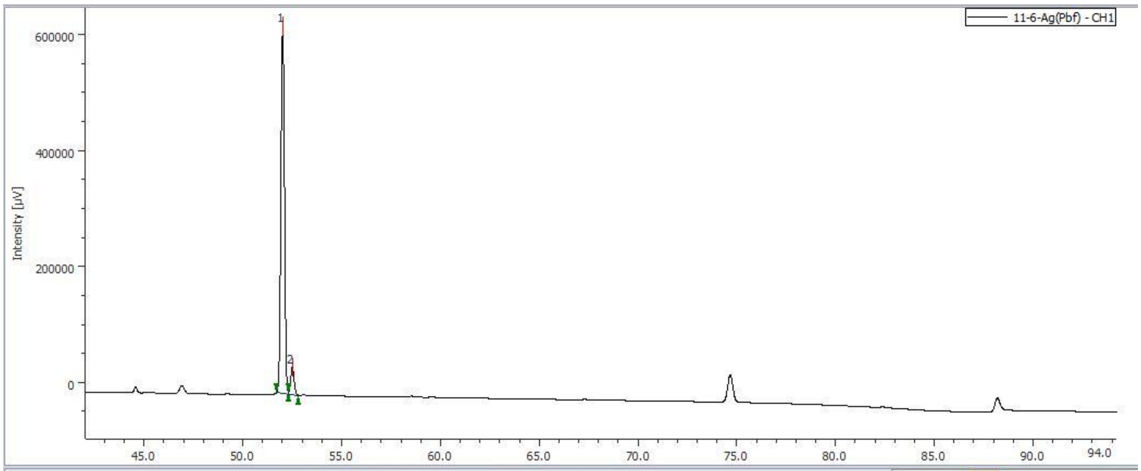

ピーク情報: 11-6-Ag(Pbf)

#	ピーク名	CH	tR	面積	高さ	面積%	高さ%	定量値	NTP	分離度	シンメトリー係数	警告
1	Unknown	1	52.008	7933269	632548	93.452	93.134	N/A	384108	1.484	0.989	
2	Unknown	1	52.492	555829	46635	6.548	6.866	N/A	437137	N/A	N/A	

Cbz-Phe-Val-^{t}Leu-OH (**2bp**)

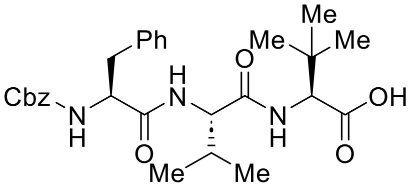

^{1}H NMR (391.8 MHz, CD_3OD): δ 8.18-8.25 (m, 0.7H), 8.10-8.17 (m, 0.7H), 7.17-7.29 (m, 10H), 4.95-5.07 (m, 2H), 4.44-4.56 (m, 1H), 4.36-4.43 (m, 1H), 4.28-4.34 (m, 1H), 3.09 (dd, J = 4.5 Hz, J = 13.9 Hz, 1H), 2.82 (dd, J = 9.9 Hz, J = 13.9 Hz, 1H), 1.94-2.10 (m, 1H), 1.01 (s, 9H), 0.92 (d, J = 6.4 Hz, 6H);

^{13}C NMR (98.5 MHz, CD_3OD): δ 174.2, 174.0, 173.6, 158.2, 138.6, 138.1, 130.4, 129.4, 128.8, 128.6, 127.6, 67.5, 62.0, 60.0, 59.9, 57.6, 39.1, 35.0, 32.3, 27.2, 19.7, 19.0;

HRMS (ESI) *m/z*: $[M+Na]^+$ calcd. for $C_{28}H_{37}N_3NaO_6$, 534.2575; found 534.2581;

Analytical HPLC: t_R = 48.1 min (LLL isomer), 49.3 min (LDL isomer), method B.

Epimerization level of peptide coupling: <1% (using PTC **1b** with 5.3% epi. level)

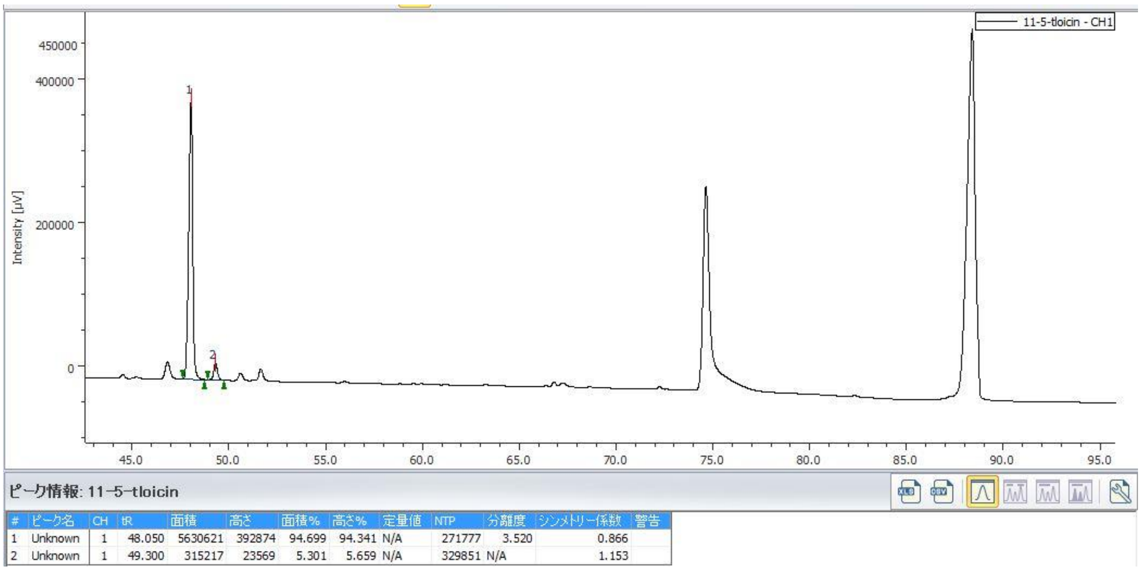

#	ピーク名	CH	tR	面積	高さ	面積%	高さ%	定量値	NTP	分離度	シンメトリー係数	警告
1	Unknown	1	48.050	5630621	392874	94.699	94.341	N/A	271777	3.520	0.866	
2	Unknown	1	49.300	315217	23569	5.301	5.659	N/A	329851	N/A	1.153	

Fmoc-Phe-Val-Val-OH (**2cb**)

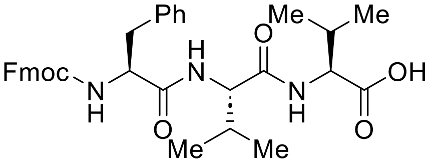

^{1}H NMR (600 MHz, $CDCl_3$): δ 7.75 (d, *J* = 7.2 Hz, 2H), 7.50 (d, *J* = 6.6 Hz, 2H), 7.35-7.45 (m, 2H), 7.21-7.31 (m, 7H), 7.11-7.20 (br, 1H), 6.80-6.90 (br, 0.3H), 6.60-6.70 (br, 0.4H), 5.40-5.60 (br, 0.5H), 4.15-4.52 (m, 6H), 3.00-3.20 (br, 2H), 2.18-2.30 (m, 1H), 2.05-2.16 (m, 1H), 0.80-0.97(m, 12H);
^{13}C NMR (150.9 MHz, DMSO-d_6): δ 172.9, 171.5, 171.2, 155.9, 143.9, 140.8, 138.3, 129.4, 128.1, 127.8, 127.2, 126.4, 125.5, 125.4, 120.2, 65.8, 57.5, 56.2, 46.7, 37.5, 31.0, 29.9, 19.3, 19.2, 18.2;
HRMS (ESI) *m/z*: $[M+Na]^+$ calcd. for $C_{34}H_{39}N_3NaO_6$, 608.2731; found 608.2738;
Analytical HPLC: t_R = 53.5 min (LLL isomer), 54.3 min (LDL isomer), method B.
Epimerization level of peptide coupling: <1% (using PTC **1c** with 13.2% epi. level)

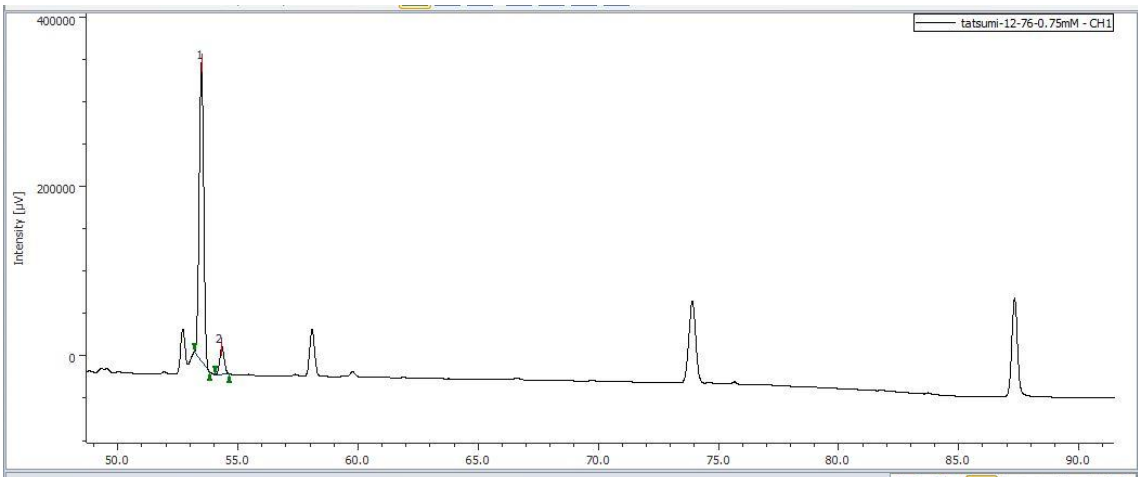

ピーク情報: tatsumi-12-76-0.75mM

#	ピーク名	CH	tR	面積	高さ	面積%	高さ%	定量値	NTP	分離度	シンメトリー係数	警告
1	Unknown	1	53.483	4622278	352326	91.890	91.764	N/A	366499	2.443	0.984	
2	Unknown	1	54.333	407935	31623	8.110	8.236	N/A	398329	N/A	1.060	

Boc-Phe-Val-Val-OH (**2db**)

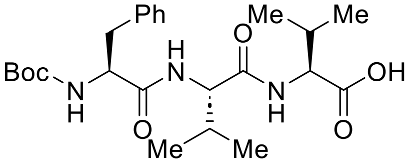

^{1}H NMR (500 MHz, CD_3OD): δ 8.22 (d, J = 8.0 Hz, 0.2H), 7.93 (d, J = 8.6 Hz, 0.4H), 7.17-7.24 (m, 5H), 7.18 (d, J = 6.9 Hz, 0.3H), 4.30-4.35 (m, 3H), 3.10 (dd, J = 4.6 Hz, J = 13.7 Hz, 1H), 2.79 (dd, J = 9.7 Hz, J = 13.7 Hz, 1H), 2.12-2.24 (m, 1H), 2.00-2.10 (m, 1H), 1.34 (s, 9H), 0.90-1.02 (m, 12H);

^{13}C NMR (125.8 MHz, CD_3OD): δ174.5, 174.2, 173.5, 157.6, 138.7, 130.3, 129.3, 127.6, 80.6, 59.7, 59.0, 57.2, 39.0, 32.4, 31.7, 28.6, 19.7, 19.6, 18.8, 18.4;

HRMS (ESI) m/z: $[M+Na]^+$ calcd. for $C_{24}H_{37}N_3NaO_6$, 486.2575; found 486.2577;

Analytical HPLC: t_R = 44.2 min (LLL isomer), 45.2 min (LDL isomer), method B.

Epimerization level of peptide coupling: <1% (using PTC **1d** with 5.1% epi. level)

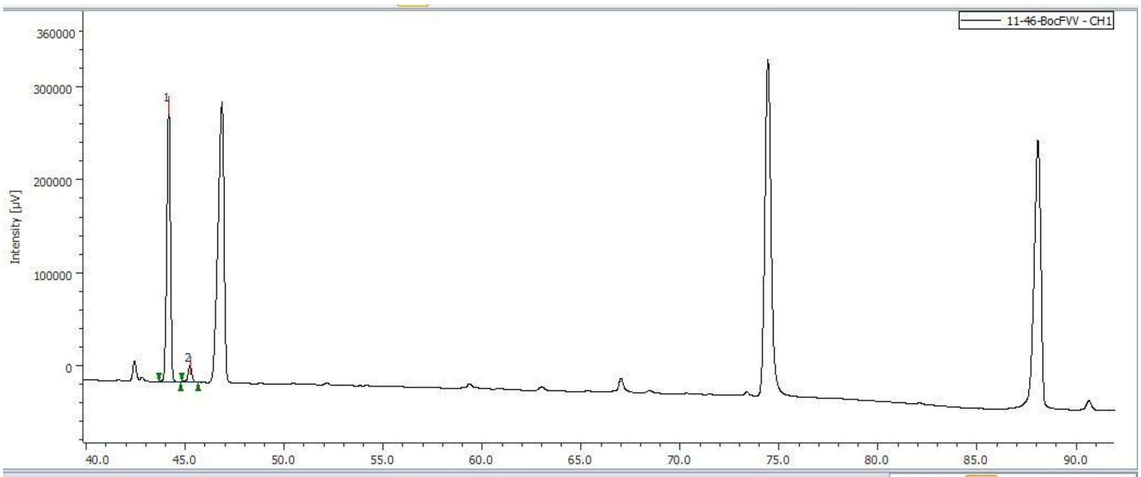

ピーク情報: 11−46−BocFVV

#	ピーク名	CH	tR	面積	高さ	面積%	高さ%	定量値	NTP	分離度	シンメトリー係数	警告
1	Unknown	1	44.167	3797915	296976	94.198	94.329	N/A	271382	3.167	0.898	
2	Unknown	1	45.233	233942	17853	5.802	5.671	N/A	289600	N/A	0.982	

Cbz-Phe-Phe-Val-OH (**2ab**)

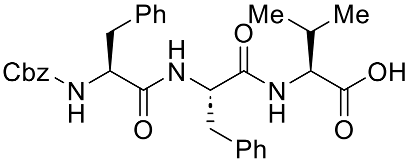

^{1}H NMR (391.8 MHz, CD_3OD): δ 7.98-8.26 (m, 1H), 7.12-7.31 (m, 15H), 4.94-5.12 (m, 2H), 4.68-4.78 (br, 1H), 4.28-4.40 (m, 2H), 3.13 (dd, J = 5.4 Hz, J = 13.9 Hz, 1H), 3.00 (dd, J = 4.9 Hz, J = 13.9 Hz, 1H), 2.91 (dd, J = 8.5 Hz, J = 13.9 Hz, 1H), 2.72 (dd, J = 9.9 Hz, J = 13.9 Hz, 1H), 2.14-2.20 (m, 1H), 0.94 (d, J = 6.7 Hz, 6H);

^{13}C NMR (150.9 MHz, DMSO-d_6): δ 173.0, 171.5, 171.3, 155.9, 138.2, 137.8, 137.2, 129.5, 129.3, 128.5, 128.2, 127.8, 127.6, 126.4, 126.3, 65.4, 57.4, 56.3, 53.7, 37.7, 37.6, 30.2, 19.3, 18.2;

HRMS (ESI) *m/z*: $[M+Na]^+$ calcd. for $C_{31}H_{35}N_3NaO_6$, 568.2418; found 568.2424;

Analytical HPLC: t_R = 49.3 min (LLL isomer), 50.7 min (LDL isomer), method B.

Epimerization level of peptide coupling: <1% (using PTC **1a** with 1.2% epi. level)

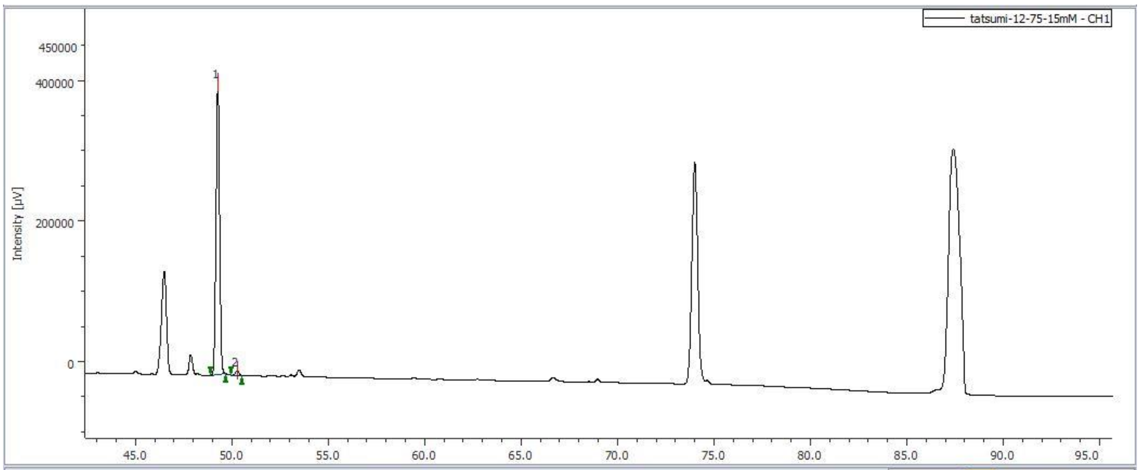

#	ピーク名	CH	tR	面積	高さ	面積%	高さ%	定量値	NTP	分離度	シンメトリー係数	警告
1	Unknown	1	49.267	5345151	414399	98.532	98.426	N/A	332130	3.017	1.009	
2	Unknown	1	50.275	79613	6625	1.468	1.574	N/A	375428	N/A	1.003	

Cbz-Phe-His(Trt)-Val-OH (**2eb**)

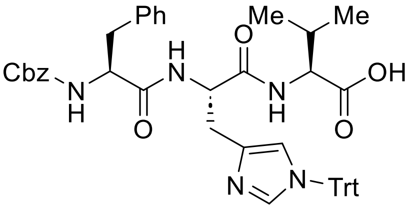

^{1}H NMR (600 MHz, DMSO-d_6): δ 8.34 (d, J = 7.8 Hz, 1H), 7.92 (d, J = 8.4 Hz, 1H), 7.39 (d, J = 8.4 Hz, 1H), 7.18-7.39 (m, 20H), 7.00-7.10 (m, 6H), 6.75-6.85 (br s, 1H), 4.90 (d, J = 12.6 Hz, 1H), 4.82 (d, J = 12.6 Hz, 1H), 4.60-4.65 (m, 1H), 4.15-4.30 (m, 2H), 2.89-2.98 (m, 2H), 2.80 (dd, J = 8.8, 14.8 Hz, 1H), 2.67 (dd, J = 10.9, 13.8 Hz, 1H), 2.01-2.11 (m, 1H), 0.75-0.90 (m, 6H);

^{13}C NMR (150.9 MHz, DMSO-d_6): δ 172.9, 171.4, 171.2, 155.9, 142.3, 138.3, 137.6, 137.1, 129.4, 129.3, 128.4, 128.4, 128.2, 127.8, 127.5, 126.4, 119.2, 74.5, 65.3, 57.3, 56.4, 52.7, 37.5, 30.1, 19.2, 18.0;

HRMS (ESI) m/z: [M+Na]$^+$ calcd. for $C_{47}H_{47}N_5NaO_6$, 800.3419; found 800.3434;

Analytical HPLC: t_R = 52.6 min (mixture of LLL and LDL isomers),

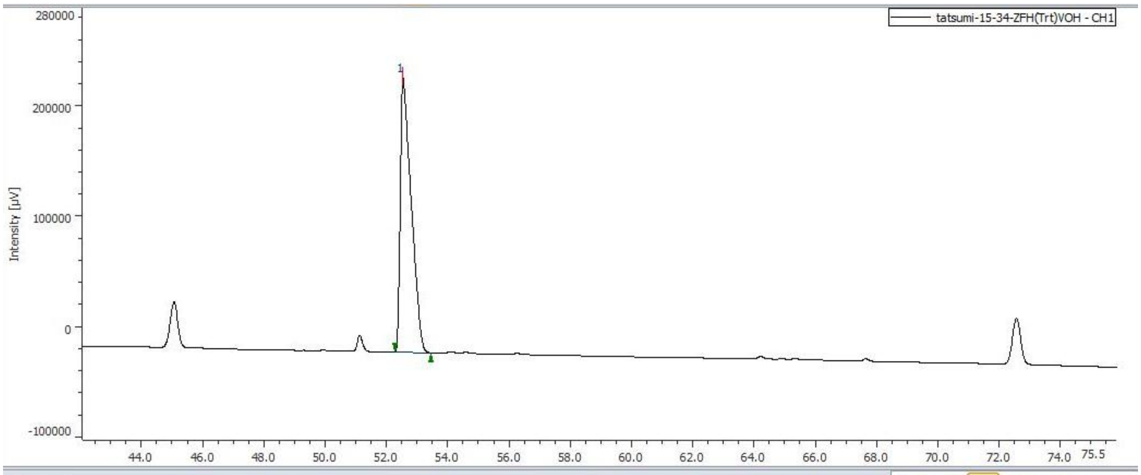

ピーク情報: tatsumi-15-34-ZFH(Trt)VOH

#	ピーク名	CH	tR	面積	高さ	面積%	高さ%	定量値	NTP	分離度	シンメトリー係数	警告
1	Unknown	1	52.525	6126834	249338	100.000	100.000	N/A	99991	N/A	2.200	

Cbz-Phe-Cys(Trt)-Val-OH (**2fb**)

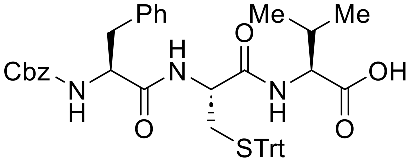

^{1}H NMR (600 MHz, $CDCl_3$): δ 7.10-7.40 (m, 25H), 6.70-6.80 (br, 1H), 6.21-6.31 (br, 1H), 5.15-5.25 (br, 1H), 5.04 (d, J = 12.6 Hz, 1H), 4.94 (d, J = 12.6 Hz, 1H), 4.30-4.45 (m, 2H), 4.01-4.08 (m, 1H), 2.90-3.15 (m, 2H), 2.72-2.82 (m, 1H), 2.46 (dd, J = 5.4 Hz, J = 13.2 Hz, 1H), 2.20-2.30 (m, 1H), 0.75-1.01 (m, 6H);

^{13}C NMR (150.9 MHz, DMSO-d_6): δ 172.6, 171.6, 169.8, 156.0, 144.4, 138.2, 137.1, 129.4, 129.2, 128.4, 128.2, 128.1, 127.9, 127.8, 127.7, 127.5, 126.9, 126.8, 126.4, 65.9, 65.4, 57.3, 56.3, 51.7, 37.5, 33.8, 30.3, 19.1, 18.0;

HRMS (ESI) *m/z*: $[M+Na]^+$ calcd. for $C_{44}H_{45}N_3NaO_6S$, 766.2921; found 766.2922;

Analytical HPLC: t_R = 62.2 min (LLL isomer), 63.4 min (LDL isomer), method B.

Epimerization level of peptide coupling: 1.4% (using PTC **1f** with 1.3% epi. level)

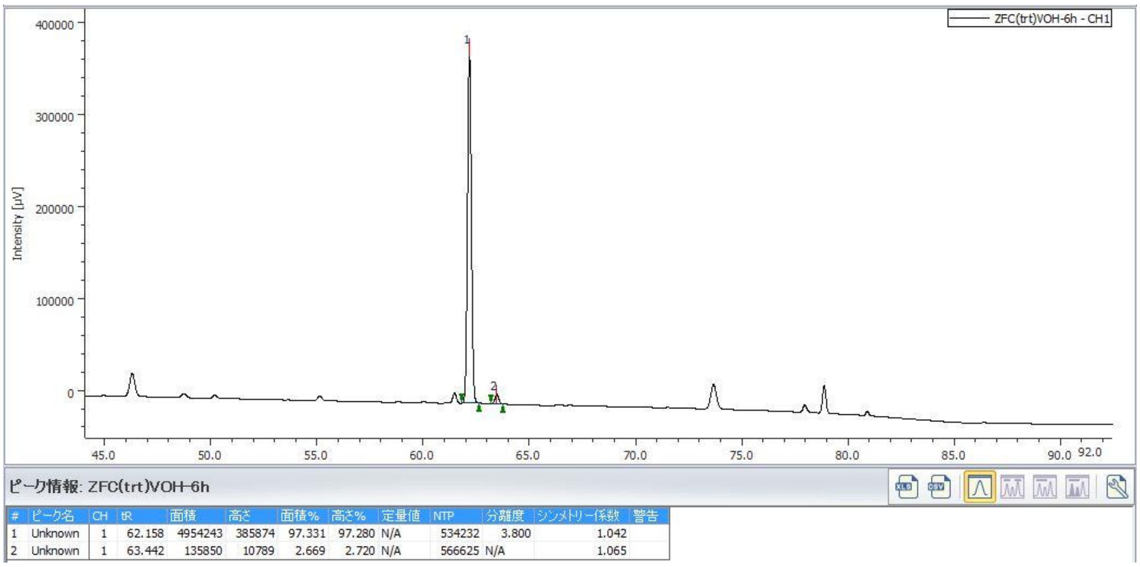

Cbz-Phe-Pro-Val-OH (**2gb**)

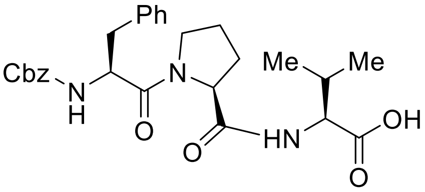

^{1}H NMR (600 MHz, DMSO-d_6): δ 7.95 (d, J = 8.4 Hz, 1H), 7.64 (d, J = 8.4 Hz, 1H), 7.19-7.34 (m, 10H), 4.85-4.99 (m, 2H), 4.48-4.52 (m, 1H), 4.35-4.44 (m, 1H), 4.10-4.16 (m, 1H), 3.57-3.70 (m, 2H), 2.93 (dd, J = 3.5, 14.0 Hz, 1H), 2.74 (dd, J = 10.4, 14.0 Hz, 1H), 1.75-2.15 (m, 5H), 0.81-0.92 (m, 6H);

^{13}C NMR (150.9 MHz, DMSO-d_6): δ 173.2, 171.6, 170.3, 156.1, 138.1, 137.2, 129.5, 128.5, 128.3, 127.9, 127.7, 126.5, 65.4, 59.3, 57.3, 54.6, 46.9, 36.5, 30.3, 28.9, 24.7, 19.4, 18.2;

HRMS (ESI) *m/z*: $[M+Na]^+$ calcd. for $C_{27}H_{33}N_3NaO_6$, 518.2261; found 518.2269;

Analytical HPLC: t_R = 43.4 min (LLL isomer), method B.

Epimerization level of peptide coupling: <1% (using PTC **1g** with 0.5% epi. level)

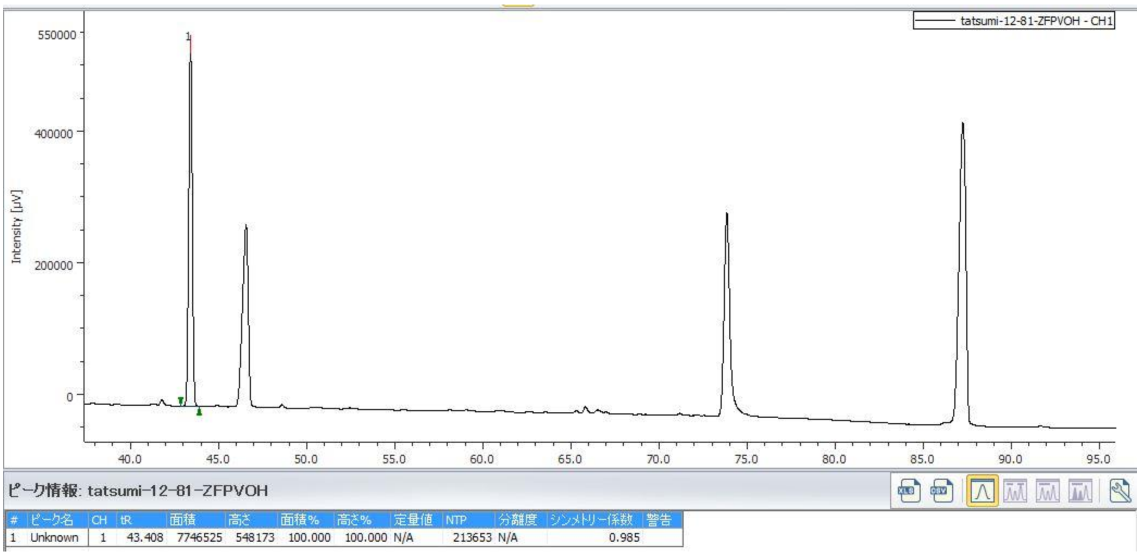

Cbz-Phe-Val-Ala-Pro-OH (**2br**)

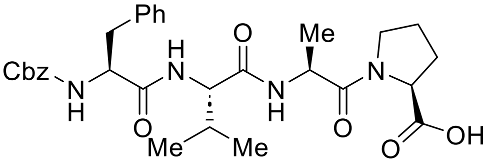

^{1}H NMR (500 MHz, CD_3OD): δ 8.31 (d, J = 5.7 Hz, 1H), 7.89 (d, J = 8.6 Hz, 1H), 7.19-7.31 (m, 11H), 5.00 (s, 2H), 4.48-4.60 (m, 1H), 4.36-4.48 (m, 2H), 4.16-4.23 (m, 1H), 3.73-3.84 (m, 1H), 3.58-3.66 (m, 1H), 3.11 (dd, J = 5.2 Hz, J = 13.7 Hz, 1H), 2.83 (dd, J = 9.7 Hz, J = 13.7 Hz, 1H), 2.18-2.30 (m, 1H), 1.90-2.13 (m, 4H), 1.34 (d, J = 6.9 Hz, 3H), 0.92 (d, J = 6.9 Hz, 3H), 0.90 (d, J = 6.3 Hz, 3H);
^{13}C NMR (125.8 MHz, DMSO-d_6): δ 173.4, 171.6, 170.4, 170.4, 156.0, 138.2, 137.1, 129.3, 128.4, 128.2, 127.8, 127.5, 126.4, 65.3, 58.6, 57.3, 56.2, 46.5, 46.3, 37.4, 31.0, 28.7, 24.7, 19.2, 18.1, 16.7;
HRMS (ESI) *m/z*: $[M+Na]^+$ calcd. for $C_{30}H_{38}N_4NaO_7$, 589.2633; found 589.2647;
Analytical HPLC: t_R = 41.2 min (LLLL isomer), 42.2 min (LDLL isomer), method B.
Epimerization level of peptide coupling: <1% (using PTC **1b** with 5.2% epi. level)

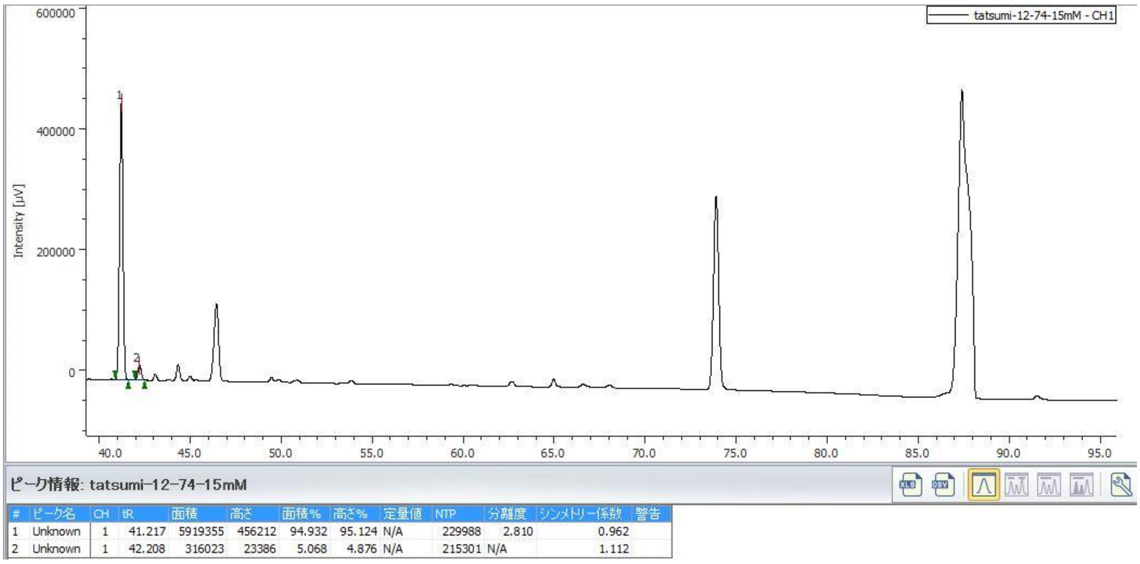

#	ピーク名	CH	tR	面積	高さ	面積%	高さ%	定量値	NTP	分離度	シンメトリー係数	警告
1	Unknown	1	41.217	5919355	456212	94.932	95.124	N/A	229988	2.810	0.962	
2	Unknown	1	42.208	316023	23386	5.068	4.876	N/A	215301	N/A	1.112	

10-3 Synthetic intermediates for DSIP

Boc-Trp-Ala-OH (**Boc-WA**)

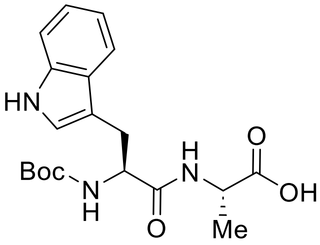

^{1}H NMR (500 MHz, CD_3OD): δ 10.27-10.34 (br, 0.5H), 7.59 (d, *J* = 7.4 Hz, 1H), 7.30 (d, *J* = 8.0 Hz, 1H), 7.11 (s, 1H), 7.03-7.09 (m, 1H), 6.92-7.02 (m, 1H), 6.45 (d, *J* = 8.0 Hz, 0.4H), 4.28-4.44 (m, 2H), 3.21-3.30 (m, 1H), 3.02-3.08 (m, 1H), 1.34 (s, 9H), 1.16-1.25 (m, 3H);
^{13}C NMR (150.9 MHz, DMSO-d_6): δ 174.3, 172.0, 155.3, 136.2, 127.6, 123.9, 121.0, 118.7, 118.3, 111.4, 110.4, 78.1, 55.0, 47.7, 28.3, 27.9, 17.5;
HRMS (ESI) *m/z*: $[M+Na]^+$ calcd. for $C_{19}H_{25}N_3NaO_5$, 398.1686; found 398.1710.
LC-MS: t_R = 16.9 min, method G.

Boc-Trp-Ala-Gly-OH (**Boc-WAG**)

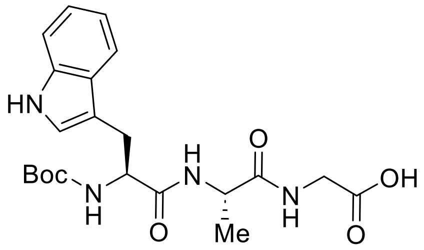

^{1}H NMR (600 MHz, $CDCl_3$): δ 8.36-8.45 (br, 1H), 7.62 (d, *J* = 7.8 Hz, 1H), 7.34 (d, *J* = 8.4 Hz, 1H), 7.19 (t, *J* = 7.2 Hz, 1H), 7.11 (t, *J* = 7.2 Hz, 1H), 7.06 (s, 1H), 6.50-6.64 (br, 0.6H), 6.36-6.42 (br, 0.5H), 5.20-5.32 (br, 1H), 4.40-4.60 (m, 2H), 3.87-3.95 (m, 1H), 3.50-3.62 (m, 1H), 3.22 (d, *J* = 7.2 Hz, 2H), 1.43 (s, 9H), 1.22 (d, *J* = 6.6 Hz, 3H);

^{13}C NMR (150.9 MHz, DMSO-d_6): δ 172.6, 171.7, 171.2, 155.4, 136.2, 127.6, 123.9, 121.0, 118.7, 118.3, 111.4, 110.4, 78.2, 55.3, 48.1, 28.3, 27.7, 18.6;

HRMS (ESI) *m/z*: $[M+Na]^+$ calcd. for $C_{21}H_{28}N_4NaO_6$, 455.1901; found, 455.1905;

LC-MS: t_R = 15.5 min, method G.

Fmoc-Gly-Asp(^{t}Bu)-OH (**Fmoc-GD(^{t}Bu)**)

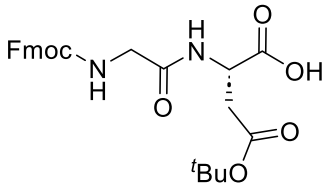

^{1}H NMR (391.8 MHz, CD_3OD): δ 7.79 (d, *J* = 6.5 Hz, 2H), 7.66 (d, *J* = 7.2 Hz, 2H), 7.38 (t, *J* = 7.6 Hz, 2H), 7.31 (t, *J* = 7.2 Hz, 2H), 4.72 (t, *J* = 5.8 Hz, 1H), 4.28-4.42 (m, 2H), 4.16-4.28 (m, 1H), 3.78-3.84 (m, 2H), 2.75 (d, *J* = 5.8 Hz, 2H), 1.40 (s, 9H);

^{13}C NMR (150.9 MHz, DMSO-d_6): δ 172.3, 169.4, 169.1, 156.6, 144.0, 140.9, 127.9, 127.3, 125.4, 120.3, 80.6, 65.9, 48.7, 46.8, 43.4, 37.4, 27.8;

HRMS (ESI) *m/z*: $[M+Na]^+$ calcd. for $C_{25}H_{28}N_2NaO_7$, 491.1789; found 491.1800;

LC-MS: t_R = 20.7 min, method H.

Fmoc-Gly-Asp(^{t}Bu)-Ala-OH (**Fmoc-GD(^{t}Bu)A**)

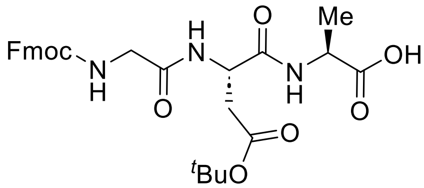

^{1}H NMR (391.8 MHz, CD_3OD): δ 7.79 (d, *J* = 7.2 Hz, 2H), 7.66 (d, *J* = 7.6 Hz, 2H), 7.38 (t, *J* = 7.2 Hz, 2H), 7.30 (t, *J* = 7.2 Hz, 2H), 4.79 (dd, *J* = 4.9 Hz, *J* = 7.6 Hz, 1H), 4.31-4.40 (m, 3H), 4.16-4.25 (m, 1H), 3.79 (s, 2H), 2.77 (dd, *J* = 4.9 Hz, *J* = 16.1 Hz, 1H), 2.63 (dd, *J* = 7.6 Hz, *J* = 16.1 Hz, 1H), 1. (m, 12H);
^{13}C NMR (125.8 MHz, $CDCl_3$): δ175.0, 171.2, 170.7, 169.8, 157.1, 143.7, 141.3, 127.8, 127.2, 125.1, 120.0, 82.3, 67.5, 49.8, 48.7, 47.0, 44.4, 37.3, 28.0, 17.3;
HRMS (ESI) *m/z*: [M+Na]$^+$ calcd. for $C_{28}H_{33}N_3NaO_8$, 562.2160; found 562.2167;
LC-MS: t_R = 25.2 min, method E.

Fmoc-Ser(^{t}Bu)-Gly-OH (**Fmoc-S(^{t}Bu)G**)

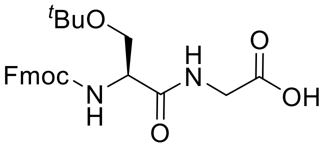

^{1}H NMR (500 MHz, $CDCl_3$): δ 7.76 (d, *J* = 7.4 Hz, 2H), 7.52-7.62 (br, 2H), 7.40 (t, *J* = 7.4 Hz, 2H), 7.31 (t, *J* = 7.4 Hz, 2H), 5.70-5.82 (br, 1H), 4.36-4.50 (br, 2H), 4.26-4.35 (br, 1H), 4.23 (t, *J* = 6.9 Hz, 1H), 4.01-4.18 (br, 2H), 3.72-3.86 (br, 1H), 3.42 (t, *J* = 8.3 Hz, 1H), 1.26 (s, 9H);
^{13}C NMR (98.5 MHz, $CDCl_3$): δ172.7, 171.1, 156.5, 143.8, 141.4, 127.9, 127.2, 125.2, 120.1, 74.6, 67.4, 61.8, 54.6, 47.2, 41.5, 27.4;
HRMS (ESI) *m/z*: [M+Na $^+$ calcd. for $C_{24}H_{28}N_2NaO_6$, 463.1840; found 463.1857;
LC-MS: t_R = 20.9 min, method G.

Fmoc-Ser(^{t}Bu)-Gly-Glu(^{t}Bu)-OH (**Fmoc-S(^{t}Bu)GE(^{t}Bu)**)

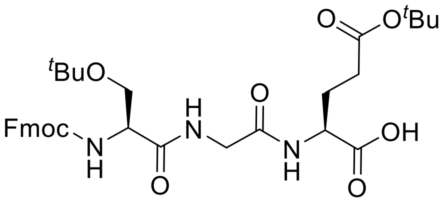

^{1}H NMR (391.8 MHz, CD_3OD): δ 8.37-8.42 (br, 0.6H), 7.99 (d, *J* = 7.6 Hz, 0.7H), 7.78 (d, *J* = 7.6 Hz, 2H), 7.66 (d, *J* = 7.2 Hz, 2H), 7.36 (t, *J* = 7.6 Hz, 2H), 7.29 (t, *J* = 7.6 Hz, 2H), 4.30-4.51 (m, 3H), 4.18-4.26 (m, 1H), 4.10-4.18 (m, 1H), 3.96-3.4.04 (m, 1H), 3.76-3.88 (m, 1H), 3.64 (d, *J* = 5.8, 2H), 2.24-2.36 (m, 2H), 2.06-2.20 (m, 1H), 1.82-1.96 (m, 1H), 1.38 (s, 9H), 1.17(s, 9H);
^{13}C NMR (150.9 MHz, DMSO-d_6): δ 173.5, 173.2, 171.6, 170.3, 169.5, 168.7, 156.2, 144.0, 143.9, 140.9, 140.8, 127.8, 127.2, 125.4, 120.3, 79.9, 73.1, 65.9, 61.8, 55.7, 51.1, 46.8, 42.1, 31.2, 27.9, 27.4, 26.7;

HRMS (ESI) *m/z*: $[M+Na]^+$ calcd. for $C_{33}H_{43}N_3NaO_9$, 648.2892; found 648.2906;

LC-MS: t_R = 23.1 min, method H.

Boc-Trp-Ala-Gly-Gly-Asp(^{t}Bu)-Ala-OH (**3**)

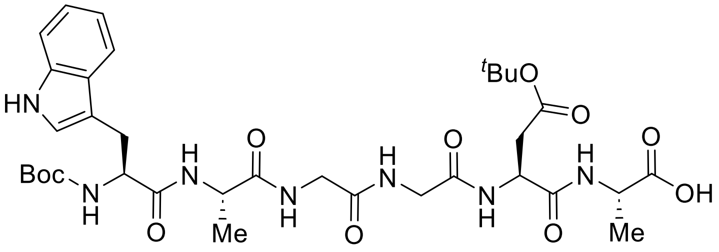

HRMS (ESI) *m/z*: $[M+Na]^+$ calcd. for $C_{34}H_{49}N_7NaO_{11}$, 754.3382; found 754.3395;

LC-MS: t_R = 21.3 min, method E.

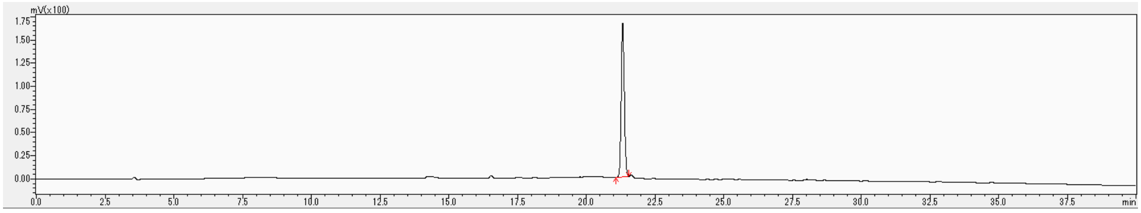

Boc-Trp-Ala-Gly-Gly-Asp(^{t}Bu)-Ala-Ser(^{t}Bu)-Gly-Glu(^{t}Bu)-OH (**4**)

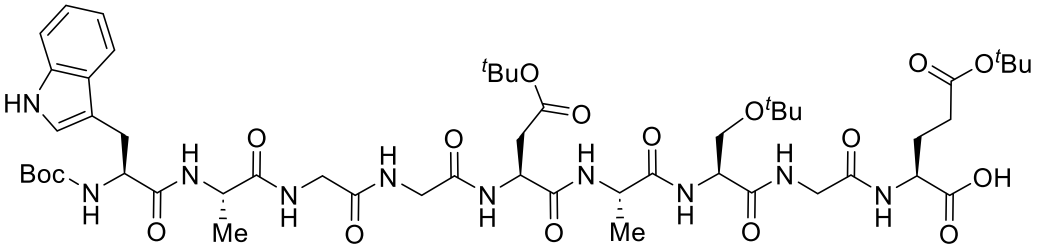

HRMS (ESI) *m/z*: $[M+Na]^+$ calcd. for $C_{52}H_{80}N_{10}NaO_{17}$, 1139.5595; found 1139.5538;

LC-MS: t_R = 24.5 min, method F.

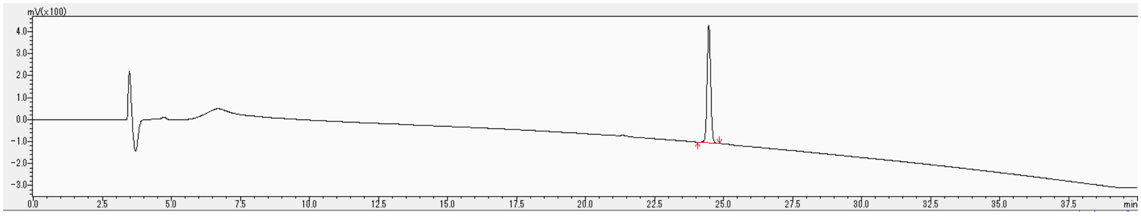

Delta-Sleep Inducing Peptide (DSIP, **WAGGDASGE**)

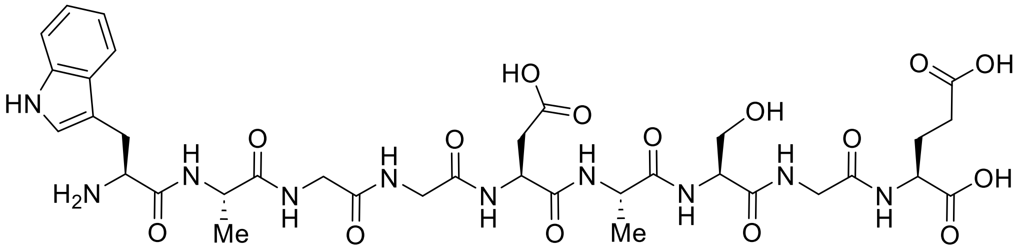

HRMS (ESI) *m/z*: $[M+Na]^+$ calcd. for $C_{35}H_{48}N_{10}NaO_{15}$, 871.3193; found 871.3201;

LC-MS: t_R = 10.6 min, method I.

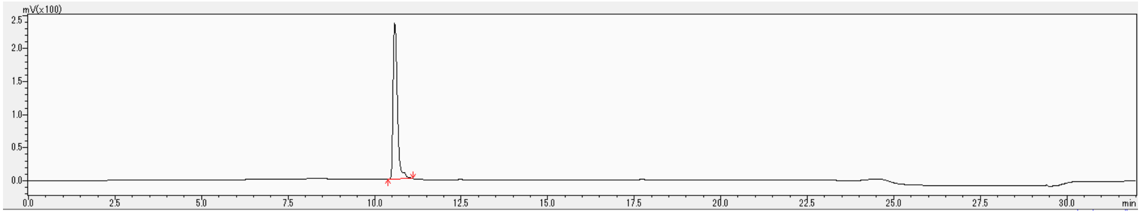

Supplementary References

(1) Matsumoto, T.; Sasamoto, K.; Hirano, R.; Oisaki, K.; Kanai, M. *Chem. Commun.* **2018**, *54*, 12222–12225.

(2) Ando, M.; Sato, N.; Nagase, T.; Nagai, K.; Ishikawa, S.; Takahashi, H.; Ohtake, N.; Ito, J.; Hirayama, M.; Mitobe, Y.; Iwaasa, H.; Gomori, A.; Matsushita, H.; Tadano, K.; Fujino, N.; Tanaka, S.; Ohe, T.; Ishihara, A.; Kanatani, A.; Fukami, T. *Bioorg. Med. Chem.* **2009**, *17*, 6106–6122.

(3) Prat, D.; Hayler, J.; Wells, A. *Green Chem.* **2014**, *16* (10), 4546–4551.

(4) Thieriet, N.; Guibé, F.; Albericio, F. *Org. Lett.* **2000**, *2*, 1815-1817.
